# MetSCORE: a molecular metric to evaluate the risk of metabolic syndrome based on serum NMR metabolomics

**DOI:** 10.1186/s12933-024-02363-3

**Published:** 2024-07-24

**Authors:** Rubén Gil-Redondo, Ricardo Conde, Chiara Bruzzone, Maria Luisa Seco, Maider Bizkarguenaga, Beatriz González-Valle, Angela de Diego, Ana Laín, Hansjörg Habisch, Christoph Haudum, Nicolas Verheyen, Barbara Obermayer-Pietsch, Sara Margarita, Serena Pelusi, Ignacio Verde, Nádia Oliveira, Adriana Sousa, Amaia Zabala-Letona, Aida Santos-Martin, Ana Loizaga-Iriarte, Miguel Unda-Urzaiz, Jasmin Kazenwadel, Georgy Berezhnoy, Tobias Geisler, Meinrad Gawaz, Claire Cannet, Hartmut Schäfer, Tammo Diercks, Christoph Trautwein, Arkaitz Carracedo, Tobias Madl, Luca Valenti, Manfred Spraul, Shelly C. Lu, Nieves Embade, José M. Mato, Oscar Millet

**Affiliations:** 1 Precision Medicine and Metabolism Laboratory, CIC bioGUNE, BRTA, CIBERehd, Bizkaia Technology Park, Bld. 800, 48160 Derio, Bizkaia Spain; 2 OSARTEN Kooperativa Elkartea, 20500 Arrasate-Mondragón, Spain; 3 Molecular Biology and Biochemistry, Gottfried Schatz Research Center, Medical University of Graz, Graz, Austria; 4 Department of Internal Medicine, Medical University, Graz, Austria; 5 Department of Internal Medicine, Medical University and University Heart Center, Graz, Austria; 6 Precision Medicine Lab, Biological Resource Center and Transfusion Medicine, Fondazione IRCCS Ca’ Granda Ospedale Maggiore Policlinico Milano, Milano, Italy; 7 Health Sciences Research Centre (CICS-UBI), 6200-506 Covilhã, Portugal; 8 CIC bioGUNE, BRTA, Derio, Bizkaia Spain; 9 CIBERONC, 28025 Madrid, Spain; 10 Traslational Prostate Cancer Research Lab, CIC bioGUNE-Basurto, Biocruces Bizkaia Health Research Institute, Barakaldo, Spain; 11 Department of Urology, Basurto University Hospital, 48013 Bilbao, Spain; 12 Department for Preclinical Imaging and Radiopharmacy, Werner Siemens Imaging Center, University of Tübingen, 72076 Tübingen, Germany; 13 Department of Internal Medicine III, Cardiology and Angiology, University Hospital Tübingen, 72076 Tübingen, Germany; 14 Bruker Biospin GmbH, Rudolf-Plank-Str. 23, 76275 Ettlingen, Germany; 15 NMR Platform, CIC bioGUNE, BRTA, Derio, Bizkaia Spain; 16 Ikerbasque, Basque Foundation for Science, 48011 Bilbao, Spain; 17 Biochemistry and Molecular Biology Department, University of the Basque Country (UPV/EHU), 20018 Bilbao, Spain; 18 BioTechMed-Graz, Graz, Austria; 19 Department of Pathophysiology and Transplantation, Università degli Studi di Milano, Milano, Italy; 20 Karsh Division of Gastroenterology and Hepatology, Cedars-Sinai Medical Center, Los Angeles, CA USA; 21 CIBER Enfermedades Hepáticas y Digestivas, Madrid, Spain

**Keywords:** Metabolic syndrome, Lipoproteins, Obesity, Dyslipidemia, Diabetes, Hypertension, NMR spectroscopy, Precision medicine

## Abstract

**Background:**

Metabolic syndrome (MetS) is a cluster of medical conditions and risk factors correlating with insulin resistance that increase the risk of developing cardiometabolic health problems. The specific criteria for diagnosing MetS vary among different medical organizations but are typically based on the evaluation of abdominal obesity, high blood pressure, hyperglycemia, and dyslipidemia. A unique, quantitative and independent estimation of the risk of MetS based only on quantitative biomarkers is highly desirable for the comparison between patients and to study the individual progression of the disease in a quantitative manner.

**Methods:**

We used NMR-based metabolomics on a large cohort of donors (n = 21,323; 37.5% female) to investigate the diagnostic value of serum or serum combined with urine to estimate the MetS risk. Specifically, we have determined 41 circulating metabolites and 112 lipoprotein classes and subclasses in serum samples and this information has been integrated with metabolic profiles extracted from urine samples.

**Results:**

We have developed MetSCORE, a metabolic model of MetS that combines serum lipoprotein and metabolite information. MetSCORE discriminate patients with MetS (independently identified using the WHO criterium) from general population, with an AUROC of 0.94 (95% CI 0.920–0.952, p < 0.001). MetSCORE is also able to discriminate the intermediate phenotypes, identifying the early risk of MetS in a quantitative way and ranking individuals according to their risk of undergoing MetS (for general population) or according to the severity of the syndrome (for MetS patients).

**Conclusions:**

We believe that MetSCORE may be an insightful tool for early intervention and lifestyle modifications, potentially preventing the aggravation of metabolic syndrome.

**Supplementary Information:**

The online version contains supplementary material available at 10.1186/s12933-024-02363-3.

## Background

Metabolic syndrome (MetS) is a complex and multifaceted pathological condition, characterised by a cluster of interrelated risk factors, that include obesity, insulin resistance/impaired fasting glucose, hypertension, and dyslipidemia [1, 2]. MetS substantially increases the risk of cardiovascular disease, type 2 diabetes, and other chronic illnesses, also affecting the liver, kidneys and/or other organs [3]. The percentage of children and adolescents affected by MetS is dramatically growing and this is directly related to the percentage of adult population that will ultimately present this syndrome, with a subsequent higher risk of developing cardiovascular diseases [4].

The definition of MetS has long been a subject of debate and lacks a universal consensus within the medical community [5, 6]. Different medical organizations and expert panels have proposed slightly varying diagnostic criteria, often only reporting a combination of the involved risk factors [6–9]. The absence of a consensual and accepted definition makes it challenging to accurately assess the prevalence of MetS, conduct comparative research across studies, and establish consistent clinical guidelines for diagnosis and management. Thus, a more objective definition of MetS, based on clinical signs and efficient molecular descriptors, would be highly desirable.

Considerable efforts have been dedicated to obtaining quantitative descriptors of MetS, based on biochemical parameters [10–12] or metabolites [13–16]. Some of these models have proved useful for the early detection of MetS in pediatric population [17] or to identify potential cardiovascular risk [18]. NMR-based metabolomics, a cutting-edge discipline in the field of systems biology [19], offers a promising avenue for unraveling the metabolic intricacies of this syndrome at the molecular level [10, 11]. Recent studies, such as those exploring the association between metabolites and depressive symptoms [20], and the effects of prenatal exposure to chemical mixtures on MetS risk in children [21], emphasize the importance of integrating metabolic health assessments with broader health outcomes. These insights highlight the necessity for comprehensive models that can provide a more nuanced understanding of MetS and its multifaceted impacts on health.

In a previous study from our group, we investigated the relative contribution of each of the mentioned risk factors to MetS by analysing the urine of a large cohort of healthy donors and patients by NMR metabolomics [22]. In there, we identified a set of metabolites directly related to the presence of each of the associated clinical factors. Yet, urine alone may not be sufficient to investigate this complex syndrome at the molecular level. While this biofluid is exquisitely sensitive to glucose levels and, potentially, to hypertension markers as well, obesity and dyslipidemia are other contributing factors to MetS [23], that may not be reflected in the urine’s metabolome since they are largely associated to the concentration of lipoprotein and metabolites in blood.

NMR metabolomics of serum includes the characterization of various, highly homeostatised circulating metabolites, and furthermore offers the possibility of profiling the lipoproteins [24, 25]. Unlike traditional bulk lipid measurements, NMR spectroscopy offers unparalleled detail by characterizing lipoproteins according to their size, density, and composition [26, 27]. The analysis of a specific region of the ^1^H-NMR spectrum of serum or plasma provides quantitative information of the four types of lipids in the lipoprotein particles: phospholipid, unesterified cholesterol, cholesterol ester, and triglyceride as well as the constituting apolipoproteins.

We now have extended our previous molecular study on MetS, based on urine measurements alone, to examine serum samples from both, the previous and new donors, completing the cohort with serum samples from individuals with a high probability of developing MetS. In total a cohort of 20,662 independent donors and up to 34,773 samples of urine/serum are considered in the present analysis. The challenges of integrating the NMR information derived from different matrices was solved by unsupervised cluster analysis, leading to an optimal molecular model, MetSCORE, based on the combined information on lipoprotein and metabolites provided from serum metabolomics, that efficiently estimates the risk of undergoing MetS (AUROC 0.94 [95% CI 0.920–0.952, p < 0.001]). This model was further validated using data from an additional independent cohort including three independent sub-cohorts, comprising a total of 661 additional samples. Altogether, MetSCORE constitutes a robust and validated model for MetS, enabling healthcare professionals to develop more personalised and precise strategies for the prevention and management of MetS.

## Methods

### Cohort study

We considered a sample donor cohort (DISCOVERY) comprising over 20,000 individuals, of whom more than 14,000 have also provided urine samples. The cohort includes 5 sub-cohorts: AKRIBEA, OSARTEN, METS+, AGEPORTUGAL, and LIVER-BIBLE. The AKRIBEA and OSARTEN sub-cohorts consist of individuals from the working general population of the Basque Country. They are complemented by: LIVER-BIBLE, which consists of individuals from Milan who are part of a study on risk factors for steatotic liver disease (SLD) and other complications of MetS; MET+, which are individuals from Navarra and Madrid who have diabetes and one or more of the other risk factors; AGEPORTUGAL, a cohort belonging to a study on aging in the Portuguese population conducted in geriatric centers. For validation purposes, VALIDATION cohort includes three additional sub-cohorts that were not employed in the model’s build-up: BPH, TÜBINGEN and BIOPERSMED. A complete description of the DISCOVERY and VALIDATION cohorts and its associated sub-cohorts can be found in the Additional file 1 (Supplement S1). Tables S1–S6 summarize the data associated with each of the sub-cohorts by gender. The gender of participants was self-reported and was not considered during the selection process. Following the principles of the Declaration of Helsinki, all individuals provided informed consent for clinical research, with the consequent evaluation and approval of the corresponding ethics committees. The specific Ethics Committee are: OSARTEN (CEIC-E 16-114); AKRIBEA (CEIC-E 19-13); BPH (CEIC-E numbers 11-12, 14-14 and 19-20); LIVERBIBLE (Fondazione IRCSS, Bambino Gesù Children’s Hospital and Palermo University Hospital, CE 125_2018bis); METS+ (CEIC-E 21-199); AGEPORTUGAL (CE-UBI-Pj-2017-012); TÜBINGEN (141/2018BO2 from Tübingen and 52/18 from Würzburg) and BIOPERSMED (Medical University of Graz, 24-224 ex 11/12). To protect patient confidentiality, all data has been double codified.

### Assignment of MetS profiles to the donors

Independent information for the associated risk factors (i.e. diabetes, obesity, dyslipidemia, and hypertension) was obtained from biochemical analysis and the general characteristics of the donors. The presence or absence of each risk of the four factors is indicated by a binary code (0 for absence and 1 for presence). The profile with no activated risk factors (0000) is named asymptomatic. We have employed the definition of the World Health Organization (WHO), referred henceforth to as WHO. The profiles that are classified as having MetS are: 1011, 1101, 1110 and 1111 (named here as *MetS_WHO*). Tables 1, S6 and S7 show a summary of the profiles and the criteria used to associate or not a risk factor to a given individual.

**Table 1:** Number of samples included in the study per gender, age group, and metabolic syndrome profile

	DISCOVERY		VALIDATION
	Total Serum	Serum with urine	Serum
	*N* = *20,662*	*N* = *14,111*	*N* = *661*
Gender: female	7821 (37.9%)	5067 (35.9%)	173 (26.2%)
Age (years)	44.7 (10.3)	43.7 (9.7)	63.7 (10.5)
Grouped age (years)			
[18,25)	424 (2.05%)	301 (2.13%)	0 (0.0%)
[25,35)	3022 (14.6%)	2233 (15.8%)	1 (0.15%)
[35,45)	6371 (30.8%)	4786 (33.9%)	6 (0.93%)
[45,55)	7488 (36.2%)	4931 (34.9%)	127 (19.7%)
[55,64)	3014 (14.6%)	1759 (12.5%)	190 (29.4%)
[64,70)	127 (0.61%)	29 (0.21%)	116 (18.0%)
[70,81)	83 (0.40%)	21 (0.15%)	167 (25.9%)
[81,105]	133 (0.64%)	51 (0.36%)	39 (6.04%)

MetS profile						
Diabetes	Obesity	Dyslipidemia	Hypertension			
(0—no; 1—yes)						
0	0	0	0	14,145 (68.5%)	10,214 (72.4%)	145 (21.9%)
0	0	0	1	1004 (4.86%)	631 (4.47%)	97 (14.7%)
0	0	1	0	1721 (8.33%)	1241 (8.79%)	70 (10.6%)
0	0	1	1	578 (2.80%)	221 (1.57%)	69 (10.4%)
0	1	0	0	1182 (5.72%)	817 (5.79%)	24 (3.63%)
0	1	0	1	361 (1.75%)	195 (1.38%)	24 (3.63%)
0	1	1	0	491 (2.38%)	338 (2.40%)	23 (3.48%)
0	1	1	1	300 (1.45%)	108 (0.77%)	40 (6.05%)
1	0	0	0	166 (0.80%)	107 (0.76%)	33 (4.99%)
1	0	0	1	139 (0.67%)	26 (0.18%)	29 (4.39%)
1	0	1	0	86 (0.42%)	40 (0.28%)	19 (2.87%)
1	0	1	1	124 (0.60%)	35 (0.25%)	35 (5.30%)
1	1	0	0	79 (0.38%)	44 (0.31%)	4 (0.61%)
1	1	0	1	91 (0.44%)	27 (0.19%)	10 (1.51%)
1	1	1	0	62 (0.30%)	31 (0.22%)	5 (0.76%)
1	1	1	1	133 (0.64%)	36 (0.26%)	34 (5.14%)
Has MetS WHO?				410 (1.98%)	129 (0.91%)	84 (12.7%)

### Sample preparation and acquisition with IVDr NMR metabolomics

Urine and serum samples were stored and manipulated according to standardised operating protocols, as previously described [28]. The ^1^H-NMR spectra were acquired on Avance IVDr 600 MHz spectrometers like other studies [22].

### NMR quantifications

Quantification of serum metabolites and lipoprotein subclasses from ^1^H-NMR spectra was performed using Bruker IVDr software, specifically B.I.Quant-PS 2.0.0 for the quantification of 41 serum metabolites in mmol/L units, and B.I.LISA (Lipoprotein Subclass Analysis) PL-5009-01/001 for the quantification of 112 serum lipoprotein classes and subclasses in mg/dL units, with the exception of particle numbers which are expressed in nmol/L and ratios which are dimensionless. The 41 quantified serum metabolites belong to the following classes: alcohols and derivatives, amines and derivatives, amino acids and derivatives, carboxylic acids, essential nutrients, keto acids and derivatives, sugars and derivatives, and sulfones. This comprehensive analysis covers the diverse types of metabolites present in the metabolome. A complete list of quantified serum metabolites and lipoproteins can be seen in Table S8. For urine samples, a pseudo-quantification of spectral regions (bins) was performed. The one-dimensional ^1^H spectrum from the NOESY experiment was segmented between 0.5 and 9.5 ppm, excluding the water region (4.7 to 5.0 ppm). The resulting spectrum was normalized by total intensity and divided into 290 fixed-width bins (0.03 ppm). Each bin is named by its ppm position, covering 0.015 ppm to the right and left. Bin intensity is the sum of the spectral point intensities within it.

### Train/test split

The DISCOVERY cohort was randomly divided (without stratification by sub-cohorts) into two sets: train (80%) and test (20%). The train set is used for variable clustering, statistical analysis, variable selection, and predictive model generation, which are subsequently evaluated on the test set.

### Metabolomic variable clustering

To facilitate analysis and avoid overrepresentation of some variables or metabolic processes, an unsupervised analysis was performed using hierarchical clustering for each variable type. The process used Spearman correlation and average as distance and linkage methods respectively. Prior to clustering, each variable was normalised by subtracting its mean and dividing by its standard deviation. Clusters were selected based on where the dendrogram is cut at a height of 0.5 for serum, and 0.85 for urine bins, since the correlation between the bins is higher (combining the metabolic effect with the effect of working with bins mentioned earlier).

### Univariate analysis

Each MetS profile was compared to the asymptomatic profile. For each metabolomic variable, a linear regression model was created with the variable as the dependent variable, while the MetS profile (i.e. each of the 16 possible states) was the independent variable, with 0000 as the reference profile. The model was adjusted for gender and age group. The coefficients associated with each profile represent the effect size of having those risk factors activated compared to having none and are expressed as the number of standard deviations. The p-values obtained for each type of variable (urine bins, serum metabolites, or serum lipoproteins) were adjusted for the False Discovery Rate (FDR) method to control for the occurrence of Type I errors due to multiple comparisons. In this study, adjusted p-values below 0.05 were considered statistically significant.

### Filtering of variables

Some variables were manually discarded. Lipoproteins based on particle number were eliminated as they have a Pearson correlation of one with Apo-B variables, although using different units. Serum metabolites related to EDTA (Ca-EDTA and K-EDTA) were also discarded. Additionally, all variables that had minimal changes (near zero variance) within both the reference group and the rest of the samples with active risk factors were also discarded. Specifically, variables were discarded if the frequency ratio of the most common value to the second most common value was greater than 19 (95/5) and the percentage of unique values was less than 10% (default values of nearZeroVar function from caret R package). All statistically significant variables with an absolute effect size of at least 0.5, as obtained from the prior univariate analysis, were selected. Variables that passed this filter are grouped according to their corresponding cluster, and only one representative of each cluster is selected, the one with the highest cumulative absolute effect size through MetS profiles. The final set of selected variables was used to discriminate between patients with *MetS_WHO* and the rest of the cohort.

### Profile visualization

A heat map was used to represent the results of the univariate analysis for the selected metabolomic variables. The colour intensity in the heat map indicates the effect size of a variable for a given profile, truncated between − 1 and 1 to enhance visualization. Asterisks inside cells highlight statistically significant differences. Both variables and profiles were organised according to the result of the dendrogram obtained by performing hierarchical clustering using Euclidean distance and complete linkage method.

### Grouping of individuals based on serum biomarkers

Self-organizing Kohonen maps were used to group individuals based on their combined profile of serum metabolites and lipoproteins (see additional information in Supplement S2 of Additional file 1). Using the representative profiles of each cell, a hierarchical grouping is created using Euclidean distance and Ward.D2 as the clustering method. The dendrogram was cut so that 6 groups were generated, which are the divisions represented within the Kohonen map.

### Predictive models for MetS

Predictive models were constructed using Orthogonal Partial Least Squares Discriminant Analysis (O-PLS-DA, see Supplement S3 of Additional file 1) method with one predictive and one orthogonal component, employing as input the variables selected from the filtering step. A logarithmic transformation is applied to input variables which are then auto scaled. The class of each individual is a binary variable indicating whether they have *MetS_WHO* or not. The predictive component (t_pred_) obtained from the model is used as measure of progression to MetS. The metrics used to evaluate the model were the area under the receiver operating characteristic (ROC) curve, sensitivity, and specificity, calculated after setting a threshold on the ROC curve based on the Youden’s index. To assess the predictive capacity of the models, they are first trained using the train set and then evaluated on the test set. Subsequently, the final model is constructed using all samples (train+test), and this model is assessed through 10 repetitions of fivefold cross-validation combined with permutation analysis (100 permutations) to estimate the statistical significance of the metrics obtained.

### Software for data analysis

All analysis were performed in R language (4.3.2) with RStudio (2023.06.2), using the following R packages: ade4 (1.7-22), caret (6.0-94), compareGroups (4.6.0), ComplexHeatmap (2.16.0), factoextra (1.0.7), ggpubr (0.6.0), ggridges (0.5.4), igraph (1.4.2), kohonen (3.0.12), tidymodels (1.1.0), tidyverse (2.0.0), and pROC (1.18.0). For O-PLS-DA models, metabom8 package (https://github.com/tkimhofer/metabom8) was used (0.4.4).

## Results

### Cohort study

To overcome the potential limitations of using urine metabolomics alone, here we explored the potential use of serum for the molecular characterization of MetS, using the serum information alone or in combination with the urine data. To that end, first we built up an ad hoc cohort (DISCOVERY), based on different existing sub-cohorts (see Materials and Methods). Overall, we obtained serum from 20,662 donors, from which we also had urine information for 14,111 donors. This cohort was designed to ensure a significant sample population in each of the 16 intermediate conditions arising from the consideration of the four main contributing risk factors: impaired fasting glucose, obesity, dyslipidemia, and hypertension (Table 1). For simplicity, we used a nomenclature for the conditions where the digits represent the four risk factors (RF_1_ RF_2_ RF_3_ RF_4_), binary coded by “1” or “0” to indicate that the given factor is present or absent in the condition (Table 1). According to this notation, a 0000 sample would originate from an apparently healthy subject without metabolic risk factors while, for instance, a sample encoded as 1110 would belong to a patient that has diabetes, obesity and dyslipidemia, but no hypertension. A quantitative definition for the inclusion criteria for each of the RF is also listed in Table S7.

### Dataset collection and grouping of variables

All the serum samples from the DISCOVERY cohort were analysed by high field NMR spectroscopy (see Materials and Methods) to obtain two different datasets: the CPMG-filtered experiment gets rid of the protein envelope and allows the quantification of up to 41 circulating metabolites (defined as the *metabo_serum* dataset). In turn, the deconvolution of the regular 1D ^1^H-NMR spectrum provided up to 112 parameters on the serum lipoproteins (*lipo_serum* dataset), including apolipoprotein, phospholipid, triglyceride, cholesterol and cholesterol ester composition and particle and sub-particle size.

**Fig. 1:**
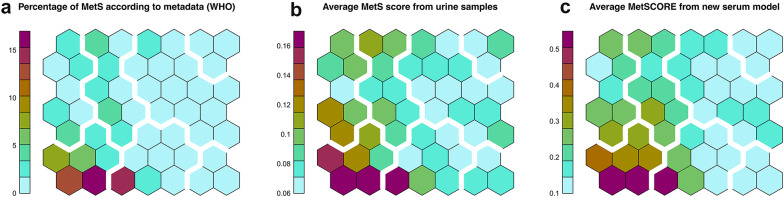
Grouping of individuals based on serum metabolomic data using a Kohonen self-organizing maps. The wide white lines separating groups of cells are the result of hierarchical clustering of the representative vectors of each cell. **a** Cells colored by the percentage of individuals with metabolic syndrome according to the metadata. This shows how serum metabolism is affected by MetS, with individuals clustering in certain regions. **b** Cells coloured according to the average metabolic syndrome score obtained from the original urine model in the previous work [22]. This score is referred as ‘MetS score’. **c** Cells coloured according to MetSCORE obtained from the serum model of this work. The scale MetSCORE is centered around a threshold of 0.5 to optimise interpretability, so MetSCORE and ‘MetS score’ are not directly comparable

We evaluated to which extent the extracted information from serum spectra is sensitive to MetS. First, Figure S1 highlights the different average concentrations for a set of metabolites and lipoparticles, when comparing people belonging to MetS_WHO (see Materials and Methods for the definition details) with the rest of the population. Figure 1a shows a grouping of individuals based on a self-organizing Kohonen map generated from information provided by serum metabolites and lipoproteins. Each cell in this type of map presents a specific pattern for its metabolites/lipoproteins and this pattern is more similar (different) the closer (farther) it is from the other cells on the plane. In Fig. 1a each cell is coloured based on the percentage of individuals classified as MetS according to the metadata associated with the samples and using the WHO definition. In turn, Fig. 1b shows the same map, but this time coloured with the score obtained with the model for discriminating MetS, based solely on metabolic information from urine samples (analysed directly as spectral bins and, for consistency, named here as *metabo_urine* dataset [22]). It can be observed that the pattern of colors obtained between the serum and *metabo_urine* datasets are very similar, suggesting that urine and serum report consistent and potentially complementary information on MetS.

Next, we attempted to combine the available datasets (*metabo_urine*, *lipo_serum* and *metabo_serum*) to refine the MetS classification molecular models, for the subset of donors were blood and urine was available (Table 1). A problem with this merging strategy is that the datasets are very different in size (112 parameters for the *lipo_serum*, 290 bins for the *metabo_urine* and 41 metabolites for the *metabo_serum*) and with different levels of cross-correlation among the variables, so a simple addition of all of them would result in a biased and meaningless result due to overrepresentation of some variables. Instead, we eliminated redundancy in the datasets using an unsupervised hierarchical clustering of the metabolomics variables. The dendrogram obtained from clustering lipoproteins (Figure S2) renders only seven groups (Figure S3): cluster 1 (LDLs 4-5) is mainly composed of LDL subclasses 4 and 5, along with Apo-B and total cholesterol; cluster 2 (HDLs 1-3 noTGs) is composed of Apo-A1 and Apo-A2 along with HDL subclasses 1, 2, and 3, except for triglycerides, which are in cluster 3 (HDLs 1-3 TGs); cluster 4 (HDLs 4 noTGs) contains HDL subclass 4; cluster 5 (VLDLs and IDLs) is mainly composed of VLDL sub-particles; cluster 6 (LDLs 1-3) is composed of LDL subclasses 1, 2, and 3; and finally, cluster 7 (LDLs 6) contains LDL-6. For serum metabolites (Figure S4), the dendrogram shows that they are quite divergent from each other, except for the groups of amino acids and ketone bodies and the cluster analysis keeps up to 28 independent variables. The dendrogram of urine bins significantly reduces complexity from 290 bins down to 15 independent variables (Figure S5). Finally, we generated a combined dataset by adding all the variables that can be extracted from serum (*metabo/lipo_serum*) and another one that resulted from the addition of the variables obtained from the urine clustering (*combined_urine/serum* dataset).

### Molecular discrimination of MetS using serum data

**Fig. 2:**
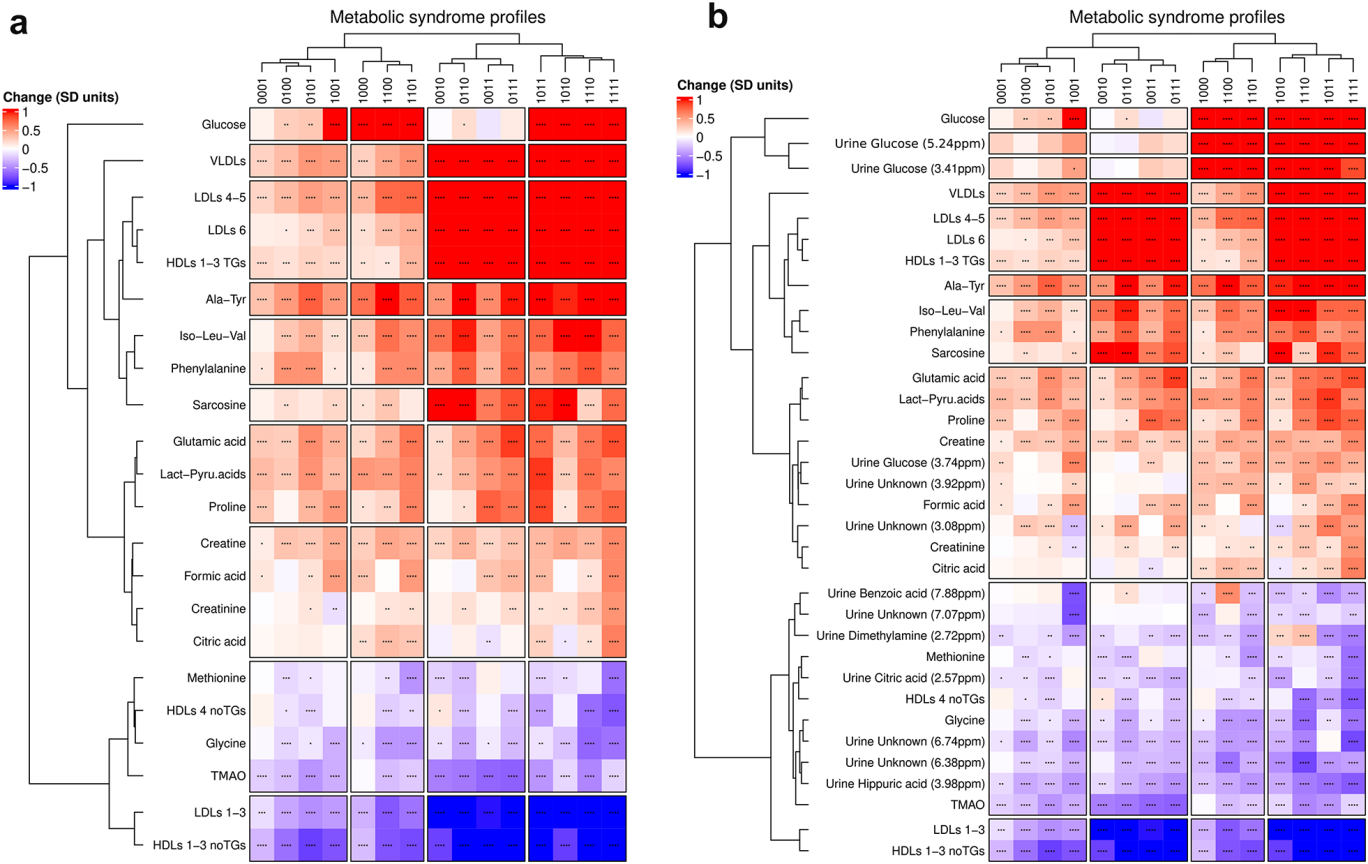
Heatmap representing the univariate analysis for each metabolic syndrome profile compared to the asymptomatic profile. Panel **a** shows the analysis for the *metabo/lipo_serum* dataset and panel **b** for the *combined_serum/urine* dataset. The colors indicate the direction of change in metabolic levels compared to the asymptomatic profile. Red indicates an increase, and blue indicates a decrease. The intensity of the colour reflects the magnitude of change in standard deviation units. Statistically significant changes are marked with asterisks (*: adjusted p-value < 0.05; **: p-value < 0.01; ***: p-value < 0.001; ****: p-value < 0.0001). Both profiles and variables are organized into clusters based on their similarities, shown in the dendrogram. These heatmaps help visualize how different metabolic parameters change in patients with metabolic syndrome. Abbreviations: 4-DEA: 4-deoxythreonic acid; TMAO: trimethylamine-N-oxide

We then compared the data obtained from individuals belonging to each condition to the equivalent data from individuals without any metabolic risk factor (samples from individuals with 0000, Fig. 2). The plot shows the most relevant variables obtained from the univariate analysis of the serum information alone (*metabo/lipo_serum,* Fig. 2a) or as combined with the urine dataset (*combined_urine/serum*, Fig. 2b). Equivalent plots are shown in the Additional file 1 for the single serum datasets (*metabo_serum* and *lipo_serum,* Figures S6 and S7). In all cases, variable selection was adjusted to account for age and gender and the conditions (in the abscise axis), and the bins/metabolites (in the ordinate axis) were sorted according to unsupervised cluster analysis. The urine bins were assigned to the contributing metabolites and up to 22/34 different metabolites/lipoprotein variables contribute to the discrimination of the conditions in the *metabo/lipo_serum* and the *combined_urine/serum respectively*. For each condition, the p-value indicates the statistical significance of the variation with respect to apparently healthy individuals (see asterisks inside the squares), while the fold change is colour-coded according to the bar legend: a red/blue value in the heatmap indicates up/down regulation of the variable.

Two main groups of profiles can be distinguished according to dyslipidemia (right versus left branches of the dendrogram), represented by an elevation of VLDL (and IDL) sub-particles, LDL subclasses 4 and 5, triglyceride-rich HDL sub-particles, and LDL subclass 6. The sub-branches further separate the profiles with diabetes from those without it. As expected, elevated glucose perfectly marks the separation of individuals with diabetes, whether in serum or urine through its associated bins. On the other hand, elevated sarcosine is also a marker of dyslipidemia in the heatmap. Additionally, subclasses 1, 2, and 3 of HDL sub-particles (except those rich in triglycerides) are reduced in dyslipidemia, along with LDL subclasses 2 and 3. Finally, amino acids such as proline and phenylalanine emerge as a potential marker for obesity and hypertension.

Remarkably, the combined serum dataset (*metabo/lipo_serum)* and the dataset that combines all data from serum and urine* (combined_urine/serum*) provided most equivalent results (Fig. 2)*.* Inclusion of urine data raises the weight of diabetes by adding urine glucose levels and reinforces the urine hypertension markers. On the other hand, inspection of the heatmaps obtained using the single datasets (*metabo_serum* and *lipo_serum,* Figures S6 & S7) revealed that they are less equilibrate, overestimating some factors such as dyslipidemia (*lipo_serum*) or diabetes conditions (*metabo_serum*), and underlining the importance of combining the two sources of information to obtain a proper molecular metric for the evolution of MetS.

### A quantitative molecular model to evaluate MetS progression

**Fig. 3:**
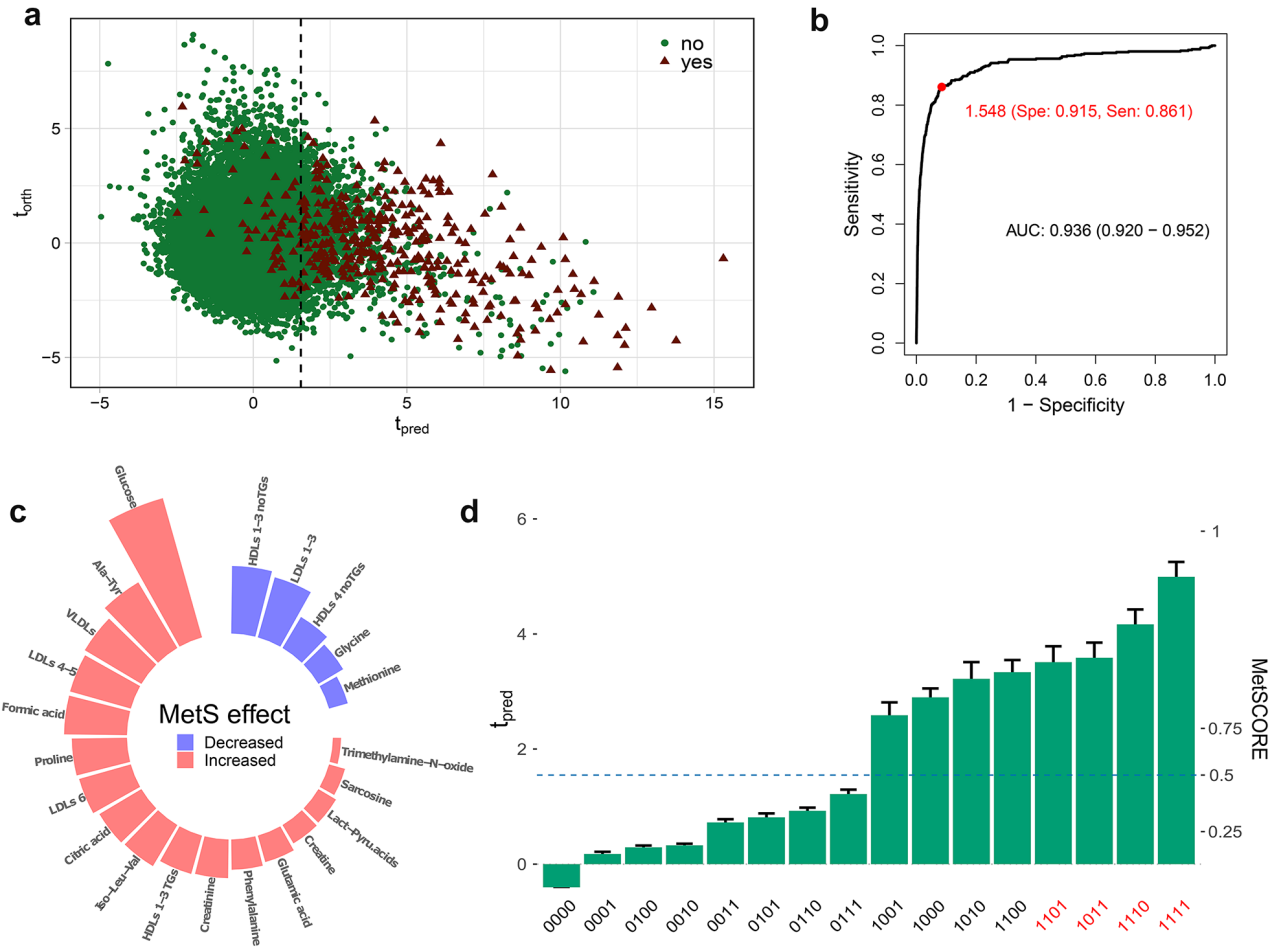
Metabolic syndrome model for the *metabo/lipo_serum* dataset based on O-PLS-DA. **a** Scores plot with the predictive component on the X axis and the orthogonal component on the Y axis. The green dots represent individuals who do not have metabolic syndrome according to their metadata and WHO criteria, while the red triangles represent those who are classified as having metabolic syndrome. This plot helps visualize the separation between individuals with and without metabolic syndrome based on their metabolic profiles. **b** ROC curve showing the area under the curve for the final model along with its 95% confidence interval. It also indicates the sensitivity and specificity for the selected cut-off based on the Youden index. The dashed horizontal line shows the threshold selected using the Youden index from the ROC curve. This curve demonstrates the model’s ability to distinguish between individuals with and without metabolic syndrome. **c** Cartoon showing the most influential variables in the model; the size of the bar indicates their relative influence, while the colour indicates whether they are elevated or not in metabolic syndrome. This visualization highlights the key biomarkers that contribute to the model’s predictive power. **d** Distribution of the predictive component averages from the O-PLS-DA model across different metabolic risk profiles. The bar plot shows the average predictive component values for various combinations of metabolic risk factors from the O-PLS-DA model. Each bar represents a unique combination of risk factors, coded in binary format on the x-axis. The left y-axis indicates the average predictive component values, while the right y-axis provides the equivalent MetSCORE for reference. The dashed blue line represents the threshold for MetS diagnosis, with scores above the line indicating a higher risk of MetS. Error bars represent the standard error of the mean for each risk profile. This plot illustrates how different combinations of metabolic risk factors contribute to the overall risk of metabolic syndrome as predicted by the model

Next, we attempted to create discrimination models for MetS, based on the available datasets. The complete predictive models, using serum or urine/serum data, were trained with O-PLS-DA to achieve a clear separation between individuals abiding *MetS_WHO* definition or not, as shown in Fig. 3 for the *metabo/lipo_serum* dataset and in Figure S8 for the *combined_urine/serum* dataset. Both models are rather equivalent but using the *metabo/lipo_serum* dataset has the advantage that only relies on a single specimen. Figure 3a shows a scores plot, with the predictive component on the X-axis and the orthogonal component on the Y-axis. A higher predictive component indicates a higher proportion of individuals with MetS. The performance of this model is quite high, with an area under the curve of 0.94 (95% CI 0.920–0.952, p < 0.001) (Fig. 3b and Table S9) when using the *metabo/lipo_serum* dataset and of 0.982 for the combined urine and serum dataset (Figure S8b). These these results are not due to overfitting (see Table S9) and the addition of the urine data to the *metabo/lipo_serum* dataset adds only marginal value at the expense of adding a second specimen to the analysis. An alternative model based only on sex, age and BMI would also discriminate for MetS (AUROC 0.92; 95% CI = 0.919–0.940). Yet, this is because most of the donors undergoing risk factors for MetS are aged individuals. When the model is tested with a healthy aged population, the *metabo/lipo_serum* model retains its capacity to discriminate MetS while the metadata-based model does not (data not shown).

As expected, the model based on the *metabo/lipo_serum* dataset has the most influential variables related to glucose and dyslipidemia, including LDLs of higher density and VLDLs increased in MetS, and decreased LDLs of lower density and HDLs not containing triglycerides (see Fig. 3c and Table S10). Elevated levels of the amino acids alanine and isoleucine are also noteworthy markers in MetS. Equivalent plots for the *combined_urine/serum* dataset model are shown in Figure S8b-c. When analysed individually, the model has a better performance in identifying diabetes (AUROC 0.928; 95% CI 0.917–0.939) than obesity (AUROC 0.730; 95% CI 0.720–0.741), dyslipidemia (AUROC 0.722; 95% CI 0.713–0.732) or hypertension (AUROC 0.717; 95% CI 0.706–0.728). This is because the gold standard is based on the WHO definition that always includes diabetes in the definition. Yet, it is important to emphasize that our metabolic model is based on the progressive change of the concentration of the metabolites, allowing to also discriminate between phenotypes that are far away from MetS (Fig. 3d), a feature that cannot be grasped with the AUROC figure of merit.

Finally, we have also generated a model that is totally uncorrelated from the molecular definitions of the associated risk factors. The serum levels of glucose and HLD/LDL particles define diabetes and dyslipidemia respectively, so we have rebuilt the model without these parameters (i.e. not considering glucose, HCLs 1-3 noTGs, HDLs4 no TGs, LDLs 6, HDLs 1-3 TGs and LDLs 4-5). The new model retains the discrimination power (AUROC 0.895, Table S11 and Figure S10), consistent with a molecular metric based on the concerted variation of many serum components (Fig. 2).

### MetSCORE, a molecular metric to classify MetS

**Fig. 4:**
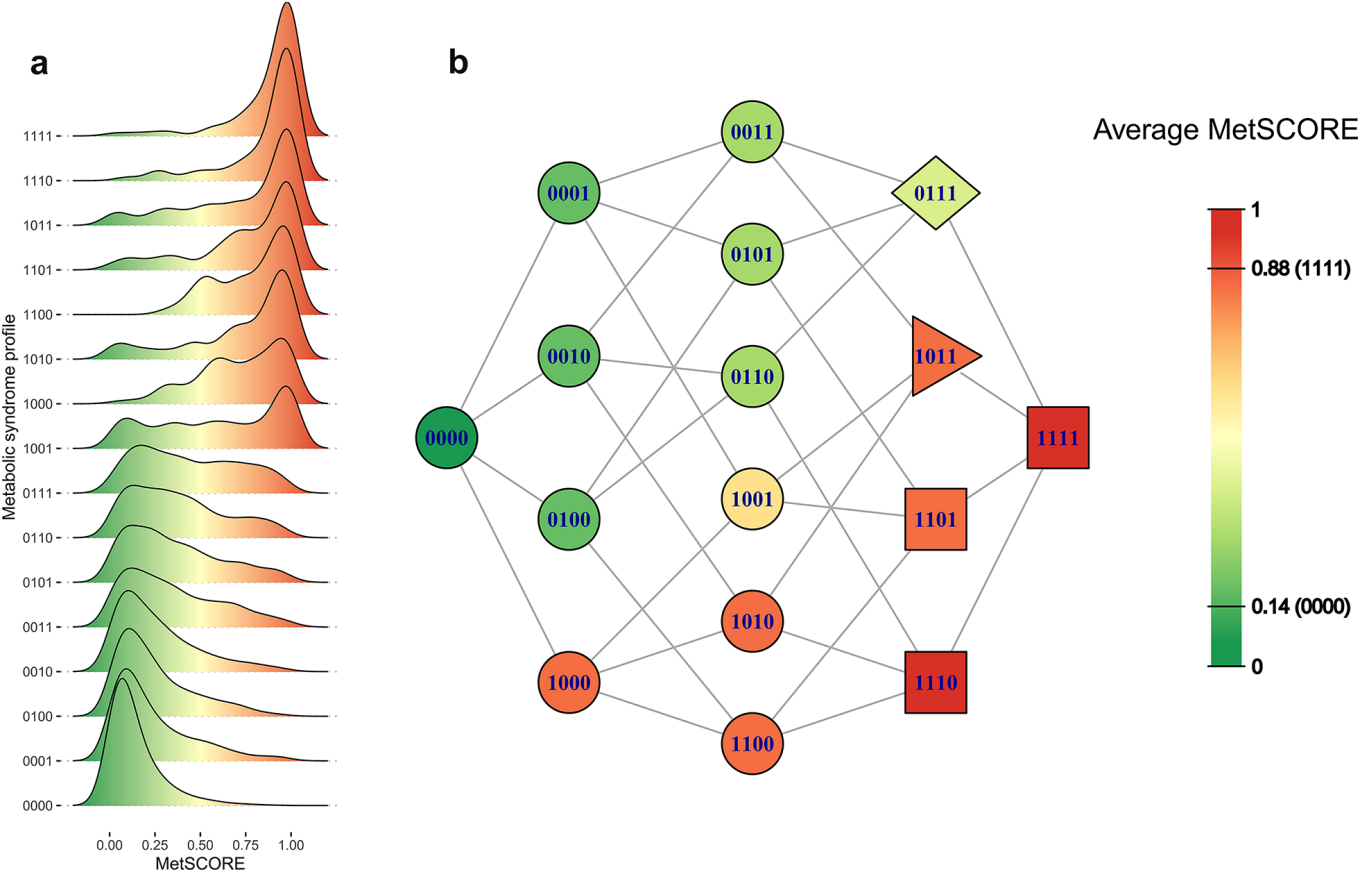
Distribution of MetSCORE values based on different profiles. **a** Ridgeline plot for MetSCORE density values by profile. The ridgeline plot shows the distribution of MetSCORE values for each profile, illustrating the continuum of metabolic states and the overlaps in boundaries when using clinical data alone. **b** Graph with the different paths that an individual can follow from the asymptomatic profile to the metabolic syndrome profile with all risk factors, colored by average MetSCORE. This graph visualizes the potential progression paths from a healthy state (0000) to a fully developed metabolic syndrome state (1111), with colours indicating the average MetSCORE along each path. The MetSCORE is normalised between 0 and 1 and the average score values for the 0000 and 1111 profiles are also indicated in the legend. Shapes within the nodes define the criteria used in various definitions of MetS: squares and triangles represent the MetS definition according to the WHO, EGIR, and AACE; squares, triangles, and rhombus represent the definition from the NCEP and Harmonized; squares and rhombus represent the definition by the IDF. This helps to understand the relative risk of developing metabolic syndrome based on different combinations of risk factors

The model derived from the *metabo/lipo_serum* dataset (including glucose and HDL/LDL particles) was used to create a score to evaluate the risk of MetS, named here MetSCORE. To that end the t_pred_ statistical distance was converted into a score by non-linear transformation through a logistic regression model (Figure S11). We adjusted the weights of each sample to ensure equal representation of both groups, thus accounting for class imbalance. This logistic regression model utilises a sigmoidal function to convert t_pred_ values into probabilities ranging between 0 and 1 and indicates the probability of developing MetS according to the analysis of the serum metabolism. Figure 4a shows the MetSCORE values for the different groups, that are now assigned to a quantitative probability of developing MetS. As expected, profiles 0000 and 1111 are the furthest apart from each other. The graph shows how the four conditions considered as MetS by the WHO are the ones populating the highest MetSCORE values, followed by the rest of the diabetes profiles without MetS.

Figure 4b shows the different paths that an individual can take from asymptomatic to complete metabolic syndrome. Each column increases the number of active factors associated with metabolic syndrome (MetS). The connections between nodes represent transitions between states by changing only one risk factor (bit) at a time. The colours of the nodes indicate the average MetSCORE of individuals with that specific profile. Shapes within the nodes signify the criteria used in various definitions of MetS: squares and triangles represent the MetS definition according to the WHO, European Group for the Study of Insulin Resistance (EGIR), and American Association of Clinical Endocrinologists (AACE); squares, triangles, and rhombus represent the definition from the National Cholesterol Education Program Adult Treatment Panel III (NCEP) and Harmonized; squares and rhombus represent the definition by the International Diabetes Federation (IDF). It can be observed that the paths in the lower part of the graph, which include diabetes, increase the score with lower steps, or in other words, they approach metabolic syndrome more readily. Incidentally, the average values of the 0000 and 1111 profiles do not match with the MetSCORE boundaries, as expected.

Finally, we also tested the effect of medication (see Supplement S4 of Additional file 1). MetSCORE is largely unsensitive to drug intake and medication raises the score only in 0.019 units.

### Validation of the model using independent cohorts

As an internal validation, the MetSCORE was recalculated 10 times, using a five-fold cross-validation combined with permutation analysis. To further validate the model, we also evaluated the risk of metabolic score in an additional validation cohort (named VALIDATION), which includes three sub-cohorts that were not included in the original build-up of the model (Tables 1 and S6). The serum model was tested on the VALIDATION cohort (n = 661). This cohort is challenging for the following reasons: (1) there is a big representation (more than 65%) of elder men (64 years old as mean age); (2) it differs from the collection site (three different European countries, two of them new); (3), some individuals were received hypertensive treatment 2 h before collection and also may not have been fasting when providing the sample; and (4) an small subgroup have been measured with a different NMR probe, which could result in minor variations. That said, the model kept a high separation power (AUROC of 0.93 as compared to 0.94 in the original model, Figure S12) and it successfully distinguished nearly all patients with MetS, with a marginal number of false-negatives, indicating that no patients diagnosed with the syndrome according to the WHO were miss-diagnosed according to our model. This highlights that, despite alterations consistent with metabolic syndrome affecting the specificity of MetSCORE, its sensitivity remains unaffected.

## Discussion

The development of quantitative methods for the precise measurement of metabolites is crucial for advancing personalised healthcare. These methods may not only facilitate early disease detection and prognosis prediction but also enable the tracking of therapeutic efficacy, ultimately paving the way for more effective, personalised, and targeted interventions in the realm of precision medicine. MetS can be easily identified by monitoring a set of basic biochemical and anthropometric parameters, but a more molecular vision of the syndrome is essential to evaluate the severity and for the disease managing. Many studies have tried to associate MetS with the measurement of molecular biomarkers including metabolites [11], lipids [29] and proteins [30] among other biomolecules. Moreover, there are many attempts to provide continuous models of MetS, based on the combined analysis of biochemical and molecular parameters [31]. Here, we have further developed these concepts for an optimised characterization of MetS, using NMR-metabolomics and lipidomics in serum to provide definition-independent molecular models that describe progression towards MetS. The best results were obtained when serum or serum combined with urine and these models not only effectively identify the current “MetS” status with a very good agreement with the *MetS_WHO* definition (AUROC 0.94 in the discrimination model) but also render quantitative information on the metabolic status at intermediate stages. Initially, a multi-class classification model was considered to identify the 16 risk profiles (from 0000 to 1111) or to create individual models determining the probability of each profile. However, it was concluded that developing a complex metabolomic model to obtain information that can be easily derived from clinical data lacked practicality. Instead, the model aims to complement the classification achievable with clinical data, providing a more continuous and nuanced understanding rather than a categorical approach with diffuse boundaries between groups. This approach enhances traditional clinical classifications with a more detailed metabolic perspective, offering better insights into the progression and risk associated with MetS. Indeed, the progression is very linear in the metabolic dimension, as shown in Fig. 4a, and we transformed the result into a metric, the MetSCORE, that can be interpreted as the probability of undergoing further medical complications. In this regard, further validation of the score is required, involving the analysis of longitudinal cohorts with access to clinical information of cardiovascular events.

We also investigated the use of different datasets in the discrimination of MetS at the molecular level. The urine dataset, as previously published, provides a very good approximation with an AUROC of 0.86 for the discrimination of the syndrome according to the WHO definition [22]. Yet, urine is difficult to quantify and very sensitive to osmolarity variations [32]. Moreover, the hydrophilic character of urine scores the model towards diabetes (represented mainly by the glucose levels in urine) to the detriment of dyslipidemia, among other risk factors. The inclusion of lipoprotein analysis from serum samples has been key to correct this bias, but it needs to be combined with additional metabolic information to produce a realistic molecular model of MetS (Fig. 1). Indeed, the serum model that combines metabolic and lipidomic data (*metabo/lipo_serum*) yields a very good discrimination of MetS, with a very similar AUROC and a very similar outcome than the urine-based model (Figs. 1c and S8), but with a much more balanced distribution of contributing metabolites/lipoproteins among the different risk factors.

Linearity of the progression towards MetS enabled the possibility of generating the MetSCORE metric to evaluate the risk of undergoing MetS. When compared to the standard definitions of the syndrome (i.e. *MetS_WHO*), the MetSCORE readout suggest that the sole addition of the risk factors, as is customary in the current definition of the syndrome, is not enough for an adequate description of the MetS risk since, at a given number of risk factors, some conditions confer a higher risk of complications than others (Fig. 4b). Thus, the molecular definition proposed here in the MetSCORE adds granularity to MetS progression and it may be helpful for managing MetS, which usually involves improving diet [33], increasing physical activity [34, 35], or taking medications to control specific risk factors like blood pressure or blood sugar or lipid levels. Moreover, an independent evaluation of MetS may also be useful in diagnosing and managing comorbidities associated to the syndrome, such as cardiovascular diseases [36], NAFLD/MASLD [37], gout, obstructive sleep apnea, kidney failure [38] or certain cancers, among other diseases.

The main clinical limitation is that MetS is already accessible by combining standard biochemical data and general characteristics of the patient. That said, we believe that a continuous definition of MetS, based only on molecular descriptors is of potential clinical value since it allows to quantify the real risk of undergoing MetS.

Medication can be a confounding factor, but our preliminary analyses on the effect of medication suggest that MetSCORE can work effectively with a broad range of concomitant drugs.

The DISCOVERY and VALIDATION cohorts used in this study are pan-European, including donors from five different European countries (Austria, Germany, Italy, Spain, and Portugal). However, the study’s composition of cohorts may be viewed as a limitation, primarily consisting of Caucasian donors and a restricted number of elderly participants. Consequently, additional validations involving more diverse populations and a broader range of age groups are strongly recommended. To partially address the concern regarding age range, we performed a subgroup analysis to evaluate MetSCORE performance across different age groups. The results, shown in Figure S13, indicate that MetSCORE maintains high performance in the < 40, 40–55, and 55–70 age groups with AUROCs of 1.00 (95% CI 0.99–1.00), 0.91 (95% CI 0.87–0.94), and 0.90 (95% CI 0.88–0.93), respectively. However, performance decreases in the > 70 age group (AUROC: 0.83, 95% CI 0.77–0.88), likely due to increased comorbidities and greater metabolic alterations typically observed in older individuals. In turn, NMR-based metabolomics is robust and, therefore, has discrimination power but the technique has limited sensitivity, targeting mainly central metabolism and abundant co-factors, so the mechanistic value of the information provided is also limited.

Another potential caveat is the practical translation to the clinic, since MetSCORE requires the use of NMR spectroscopy. Yet, the measurement of the sample requires less than 15 min of spectrometer time, and an automated algorithm can integrate signals and provide the result almost in real time. MetSCORE has a straightforward interpretation, and the standardized operating procedures include the sample collection and handling, so the method could potentially be translated into the clinical practice at a reasonable cost (i.e. less than 50 EUR/sample). Additionally, we are currently exploring the possibility of translating the measurements to a benchtop NMR spectrometer (i.e. a Fourier NMR spectrometer of 80 MHz), which could potentially be deployed in the analytical laboratories of hospitals and health centers.

## Conclusions

In summary, we provide a new metric for MetS, MetSCORE, based on the metabolomic analysis of serum samples. Our model offers a quantitative and comparable description of MetS, facilitating healthcare professionals to develop personalised strategies for the prevention and management of this complex pathology.

### Supplementary Information

Supplementary File 1 (PDF 4349 kb)

## Data Availability

The data that support this study are available from the corresponding authors upon reasonable request.

## Abbreviations

AACE: American association of clinical endocrinologists
ApoB: Apolipoprotein B
AUROC: Area under receiver operating characteristic curve
EDTA: Ethylenediaminetetraacetic acid
EGIR: European group for the study of insulin resistance
FDR: False discovery rate
HDL: High-density lipoproteins
IDF: International diabetes federation
IDL: Intermediate-density lipoproteins
IVDr: In vitro diagnostics research
LDL: Low-density lipoproteins
MetS: Metabolic syndrome
NCEP: National cholesterol education program adult treatment panel III
NMR: Nuclear magnetic resonance
O-PLS-DA: Orthogonal partial least squares discriminant analysis
RF: Risk factor
ROC: Receiver operating characteristic
SLD: Steatotic liver disease
TG: Triglycerides
VLDL: Very low-density lipoproteins
WHO: World health organization
