# Supplementary material for: MetSCORE: a molecular metric to evaluate the risk of metabolic syndrome based on serum NMR metabolomics
Source: Cardiovasc Diabetol. 2024 Jul 24;23:272. doi: 10.1186/s12933-024-02363-3 (PMC11271192; doi:10.1186/s12933-024-02363-3)
Supplement: Supplementary file 1 — (PDF 4349 kb) [file 12933_2024_2363_MOESM1_ESM.pdf]

Supplementary material for:

MetSCORE: a molecular metric to evaluate the risk of metabolic syndrome based on serum NMR metabolomics.

Rubén Gil-Redondo[1], Ricardo Conde[1], Chiara Bruzzone[1], Marisa Luisa Seco[2], Maider Bizkarguenaga[1], Beatriz González-Valle[1], Angela de Diego[1], Ana Laín[1], Hansjörg Habisch[3], Christoph Haudum[4], Nicolas Verheyen[5], Barbara Obermayer-Pietsch[4], Sara Margarita[6], Serena Pelusi[6], Ignacio Verde[7], Nádia Oliveira[7], Adriana Sousa[7], Amaia Zabala-Letona[8,9,10], Aida Santos-Martin[9,10,11], Ana Loizaga-Iriarte[9,10,11], Miguel Unda-Urzaiz[9,10,11], Jasmin Kazenwadel[12], Georgy Berezhnoy[12], Tobias Geisler[13], Meinrad Gawaz[13], Claire Cannet[14], Hartmut Schäfer[14], Tammo Diercks[15], Christoph Trautwein[13], Arkaitz Carracedo[8,9,10,16,17], Tobias Madl[3,18], Luca Valenti[6,19], Manfred Spraul[13], Shelly C Lu[20], Nieves Embade[1], José M Mato[1,21], Oscar Millet[1,21,*]

[1]Precision Medicine and Metabolism Laboratory, CIC bioGUNE, BRTA, CIBERehd, Derio, Bizkaia, Spain.
[2]OSARTEN Kooperativa Elkartea, 20500 Arrasate-Mondragón, Spain.
[3]Molecular Biology and Biochemistry, Gottfried Schatz Research Center, Medical University of Graz, Austria
[4]Department of Internal Medicine, Medical University, Graz, Austria
[5]Department of Internal Medicine, Medical University and University Heart Center, Graz, Austria
[6]Precision Medicine Lab, Biological Resource Center and Transfusion Medicine, Fondazione IRCCS Ca' Granda Ospedale Maggiore Policlinico Milano.
[7]Health Sciences Research Centre (CICS-UBI), 6200-506 Covilhã, Portugal.
[8]CIC bioGUNE, BRTA, Derio, Bizkaia, Spain.
[9]CIBERONC, Madrid 28025, Spain.
[10]Traslational prostate cancer Research lab, CIC bioGUNE-Basurto, Biocruces Bizkaia Health Research Institute, Spain
[11]Department of Urology, Basurto University Hospital, 48013, Bilbao, Spain.
[12]Werner Siemens Imaging Center, Department for Preclinical Imaging and Radiopharmacy, University of Tübingen, 72076 Tübingen, Germany.
[13]Department of Internal Medicine III, Cardiology and Angiology, University Hospital Tübingen, 72076 Tübingen, Germany.
[14]Bruker Biospin GmbH, Rudolf-Plank-Str. 23, 76275 Ettlingen, Germany.
[15]NMR Platform, CIC bioGUNE, BRTA, Derio, Bizkaia, Spain.
[16]Ikerbasque, Basque Foundation for Science, Bilbao 48011, Spain.
[17]Biochemistry and Molecular Biology Department, University of the Basque Country (UPV/EHU), Bilbao 20018, Spain.
[18]BioTechMed-Graz, Austria
[19]Department of Pathophysiology and Transplantation, Università degli Studi di Milano.
[20]Karsh Division of Gastroenterology and Hepatology, Cedars-Sinai Medical Center, Los Angeles, CA, USA.
[21]CIBER Enfermedades Hepáticas y Digestivas, Madrid, Spain
*To whom correspondence should be addressed

Oscar Millet
Precision Medicine and Metabolism Laboratory
CIC bioGUNE
Bizkaia Technology Park, Bld. 800
48160 Derio
Phone: +34 946 572 504
Fax: +34 946 572 502
e-mail: omillet@cicbiogune.es

TABLE OF CONTENTS

Supplement S1. Sub-cohorts description. 3
Supplement S2. Brief Insight: Kohonen Self-Organizing Maps. 4
Supplement S3. Rationale of employing O-PLS-DA. 5
Supplement S4. Evaluation of potential impact of medication on MetSCORE. 6
Table S1. General characteristics for the AKRIBEA sub-cohort. 7
Table S2. General characteristics for the OSARTEN sub-cohort. 8
Table S3. General characteristics for the LIVER-BIBLE sub-cohort. 9
Table S4. General characteristics for the METS+ sub-cohort. 10
Table S5. General characteristics for the AGEPORTUGAL sub-cohort. 11
Table S6. General characteristics for the Validation cohorts. 12
Table S7. Metabolic syndrome risk factors. 13
Table S8. List of metabolites and lipoproteins that are quantified in serum samples with Bruker IVDR software. 14
Table S9. Performance metrics of generated *metabo/lipo-serum* predictive models for MetS. 16
Table S10. Loading values for the predictive component of the *metabo/lipo-serum* O-PLS-DA model. 17
Table S11. Performance metrics of limited *metabo/lipo-serum* predictive models: excluding Glucose, HDLs and LDLs. 18
Figure S1. Comparison of raw quantifications (MetS_WHO vs. other). 19
Figure S2. Clustering of serum lipoproteins. 20
Figure S3. Mosaic plot with lipoprotein clusters and their components. 21
Figure S4. Clustering of serum metabolites. 22
Figure S5. Clustering of urine bins. 23
Figure S6. Heatmap representing the univariate analysis conducted for each metabolic syndrome profile compared to the asymptomatic profile for the metabo_serum dataset. 24
Figure S7. Heatmap representing the univariate analysis conducted for each metabolic syndrome profile compared to the asymptomatic profile for the lipo_serum dataset. 25
Figure S8. Metabolic syndrome model for the combined_serum/urine dataset based on O-PLS-DA combining urine and serum metabolomic data. 26
Figure S9. Projection of individuals from each metabolic syndrome profile onto the scores plot of the final O-PLS-DA metabo/lipo_serum model. 27
Figure S10. Metabolic syndrome model using the limited serum dataset: excluding Glucose, HDLs and LDLs. 28
Figure S11. A plot with the evolution of the mean quantification values from the significant parameters of the MetSCORE. 29
Figure S12. Performance of MetSCORE on the validation cohort. 30
Figure S13. ROC curves of MetSCORE performance across different age groups. 31
Supplementary references 32

Supplement S1. Sub-cohorts description.

AKRIBEA sub-cohort[1] consists of urine and serum samples from individuals from the working general population of the Basque Country. They were collected between 2019 and 2023 in overnight fasting conditions and during the annual medical test. People participating in this study were males and females between 18 and 67 years old. The only exclusion criterium was to have suffered a serious illness like cancer or ictus in the 3 months preceding the sample collection. Biochemical data, lifestyle habits, and medication were also recollected as general characteristics from all the donors. Blood serum samples were collected, processed and frozen in an interval of maximum 2 hours and 4 hours for urine samples. All samples were provided by the Basque Biobank for research (BIOEF). Ethics approval number: CEIC-E 19-13.

OSARTEN sub-cohort[2] consists of urine and serum samples from individuals from the working general population of the Basque Country. They were collected between 2017 and 2018 in overnight fasting conditions and during the annual medical test. People participating in this study were males and females between 19 and 66 years old. The only exclusion criterium was to have suffered a serious illness like cancer or ictus in the 3 months preceding the sample collection. Biochemical data, lifestyle habits, and medication were also recollected as general characteristics from all the donors. Blood serum samples were collected, processed and frozen in an interval of maximum 2 hours and 4 hours for urine samples. All samples were provided by the Basque Biobank for research (BIOEF). Ethics approval number: CEIC-E 16-114.

LIVER-BIBLE sub-cohort[3] consists of serum samples from individuals from Milan with metabolic dysfunction, who were consecutively enrolled from 2019 to 2022. These were apparently healthy blood donors, aged 40–65 years, who were selected for a comprehensive liver disease, metabolic and cardiovascular screening, owing to the presence of at least three metabolic risk abnormalities. Individuals with chronic degenerative diseases (such as advanced kidney disease, cirrhosis, or active cancer), except for well-controlled arterial hypertension, treated hypothyroidism and well-compensated type 2 diabetes (T2D) not requiring pharmacotherapy (except for metformin), were excluded from the cohort. Blood serum samples were collected, processed and frozen in an interval of maximum 2 hours and 4 hours for urine samples. Ethics approval number: CE 125_2018bis.

MET+ sub-cohort consists of serum samples from individuals from Navarra and Madrid who have diabetes and one or more of the other risk factors. Samples were collected before 2022 from donors aged 31-90 years. These samples were selected and obtained for research purposes from both the Madrid and Navarra biobanks to increase the number of serum sample cases potentially indicative of metabolic syndrome. Blood serum samples were collected, processed and frozen in an interval of maximum 2 hours and 4 hours for urine samples. Ethics approval number: CEIC-E 21-199.

AGEPORTUGAL sub-cohort[4] consists of serum and urine samples from individuals belonging to a study on aging in the Portuguese population, conducted in geriatric centres. Samples were collected between 2017 and 2019 in overnight fasting conditions at the nursing homes. Individuals included in this cohort comprehend ages between 65 and 94 years old. Sociodemographic data, cognitive examination, comorbidities, medication and cholesterol, glucose and triglycerides data were collected for all individuals. Blood serum samples were collected, processed and frozen in an interval of maximum 2 hours and 4 hours for urine samples. Individuals that suffered from cancer or ictus prior the sample collection were excluded. Ethics approval number: CE-UBI-Pj-2017–012.

BPH validation sub-cohort[5] consists of serum samples from individuals with benign prostatic hyperplasia recruited in Basurto University Hospital, samples accrued in the urology service before 2021 and managed by the Basque Biobank for Research (BIOEF). Blood serum samples were collected, processed and frozen in an interval of maximum 2 hours and 4 hours for urine samples. Ethics approval numbers: CEIC-E 11-12, 14-14 and 19-20.

BIOPERSMED validation sub-cohort[6] consists of serum samples from individuals participating in an observational study (Biomarkers for Personalized Medicine) in Graz/Austria to evaluate novel biomarkers in cardiovascular and metabolic diseases. To all individuals was obtained data regarding sociodemographic characteristics, biochemistry data and comorbidities (i.e. hypertension, diabetes, and dyslipidaemia). Blood serum samples were immediately aliquoted and stored at the biobank of the Medical University of Graz (Austria) at – 80°C. Ethics approval number: 24-224 ex 11/12.

TÜBINGEN validation sub-cohort[7] consists of serum samples from individuals participating in a study of antihypertensive medications; the patient recruitment took place in Tübingen, Germany. Since the data were kept in Würzburg, Germany, there is a second ethics vote with the number (52/18). The process of collecting urine and serum samples has been the same for all sub-cohorts, following the same Standard Operating Procedures (SOPs). Following the principles of the Declaration of Helsinki, all individuals provided informed consent for clinical research, with the consequent evaluation and approval of the corresponding ethics committees. To protect patient confidentiality, all data has been double codified. Ethics approval numbers: 141/2018BO2 and 52/18.

Supplement S2. Brief Insight: Kohonen Self-Organizing Maps.

Self-Organizing Maps (SOM), also known as Kohonen maps, are a type of artificial neural network originally created by Teuvo Kohonen in the 1980s[8,9]. It is used for unsupervised learning, and it serves as a technique for dimensional reduction, transforming high-dimensional data into a lower-dimensional representation while preserving the topological properties of the input space. Picture a network of neurons, each representing a specific region within the input space. Throughout training, SOM adjusts these neurons based on input similarity, allowing similar data points to be mapped closer together on the grid. This process yields a simplified representation of complex data, unveiling underlying patterns and relationships.

A significant advantage of Kohonen maps in clustering is their ability to organize data based on similarity without the need for labelled information. They efficiently group similar data points, aiding in data exploration and classification. Moreover, SOM offers advantages in information representation by creating a visual map that reflects the input data's structure. This visualization allows for intuitive interpretation and understanding of complex datasets, enabling researchers and analysts to identify trends, outliers, and relationships that might not be evident in the original high-dimensional data.

There are several examples of the use of SOM in literature, both in metabolomics and other scientific disciplines[10–15]. In summary, SOM serves as a powerful tool for data clustering by organizing data into similarity-based groups and providing a detailed representation of information, thus facilitating data exploration and comprehension.

Supplement S3. Rationale of employing O-PLS-DA.

Orthogonal Partial Least Squares Discriminant Analysis (O-PLS-DA)[16,17] stands as a pivotal technique in multivariate modelling, specifically addressing the interpretability and predictive complexities within datasets, particularly in fields like metabolomics. One of its key strengths lies in striking a delicate balance between interpretability and predictive performance, surpassing conventional methods like logistic regression in certain aspects.

When crafting a predictive model, interpretability emerges as a pivotal requirement. While logistic regression might seem to offer a straightforward interpretative approach, O-PLS-DA not only matches its interpretative strength but also surpasses it by intricately modelling the intricate relationship between predictors, notably metabolic variables, and the outcome, such as the presence or absence of metabolic syndrome. This is crucial given the inherent multicollinearity prevalent in metabolomic data, a challenge effectively addressed by O-PLS-DA. By disentangling the predictive variation from the uncorrelated systematic variation, O-PLS-DA excels in isolating essential metabolic components contributing to group distinctions, thereby significantly enhancing interpretability, especially in the context of intricate and interconnected metabolic data.

Moreover, what distinguishes O-PLS-DA from its non-orthogonal counterpart, PLS-DA, is its ability to segregate information. This segregation proves immensely beneficial in discriminating between various groups, allowing a sharper focus on the metabolic components driving these distinctions. Furthermore, O-PLS-DA extends beyond mere classification; it integrates a predictive element that not only discerns between classes but also provides a continuous measure, signifying progression across these classes. This feature is particularly valuable, offering insights into the nuanced changes and developments within and between groups, a facet not readily achievable through traditional classification methods.

Several examples of O-PLS-DA usage can be found in literature, particularly within the field of metabolomics[18–22]. In essence, O-PLS-DA emerges as a comprehensive and powerful tool in multivariate analysis, offering a nuanced understanding of complex relationships within datasets, especially in metabolomics, by merging interpretability with predictive capabilities and effectively handling multicollinearity to distil essential metabolic insights for classification and progression evaluation.

Supplement S4. Evaluation of potential impact of medication on MetSCORE.

To assess the potential effect of medication on MetSCORE, we initially selected all sub-cohorts with medication information records. Consequently, two new sub-cohorts were formed: one comprising individuals taking some form of medication and another consisting of individuals who have not reported taking any medication. The following table summarizes the number of individuals in each case and sub-cohort:

Original sub-cohort	Taking any medication?	
	No	Yes
**AGEPORTUGAL**	1	150
**AKRIBEA**	8796	2363
**BPH**	2	183
**LIVER-BIBLE**	660	397
**OSARTEN**	5846	2296
**TOTAL**	**15305**	**5389**

Using linear regression analysis, with MetSCORE as the dependent variable and the condition of taking medication or not as the independent variable, controlling for gender, age, and metabolic syndrome profile, we determine the average effect that taking medication has on MetSCORE. The following is a summary of the linear regression analysis, with the coefficient associated with taking medication highlighted in bold:

```
Coefficients:
                    Estimate Std. Error t value Pr(>|t|)
(Intercept)       -0.0238056  0.0059458  -4.004 6.26e-05 ***
any_medicationyes  0.0189606  0.0029065   6.523 7.03e-11 ***
metS_profile0001   0.0589418  0.0057642  10.225  < 2e-16 ***
metS_profile0010   0.1036095  0.0044468  23.300  < 2e-16 ***
metS_profile0011   0.1613858  0.0074115  21.775  < 2e-16 ***
metS_profile0100   0.1060756  0.0052133  20.347  < 2e-16 ***
metS_profile0101   0.1745135  0.0092716  18.822  < 2e-16 ***
metS_profile0110   0.2274352  0.0078692  28.902  < 2e-16 ***
metS_profile0111   0.2528301  0.0101058  25.018  < 2e-16 ***
metS_profile1000   0.5462220  0.0136538  40.005  < 2e-16 ***
metS_profile1001   0.3593208  0.0162928  22.054  < 2e-16 ***
metS_profile1010   0.5688137  0.0183530  30.993  < 2e-16 ***
metS_profile1011   0.5147069  0.0161715  31.828  < 2e-16 ***
metS_profile1100   0.6152239  0.0203433  30.242  < 2e-16 ***
metS_profile1101   0.5283312  0.0200432  26.360  < 2e-16 ***
metS_profile1110   0.6902128  0.0224807  30.702  < 2e-16 ***
metS_profile1111   0.6459815  0.0179795  35.929  < 2e-16 ***
sexmale            0.0405942  0.0025502  15.918  < 2e-16 ***
age                0.0031464  0.0001281  24.569  < 2e-16 ***
---
Signif. codes:  0 ‘***’ 0.001 ‘**’ 0.01 ‘*’ 0.05 ‘.’ 0.1 ‘ ’ 1

Residual standard error: 0.172 on 20665 degrees of freedom
  (10 observations deleted due to missingness)
Multiple R-squared:  0.3851,     Adjusted R-squared:  0.3845
F-statistic: 718.9 on 18 and 20665 DF,  p-value: < 2.2e-16
```

The positive effect on MetSCORE, while statistically significant (P-value < 7.03e-11), is very small, merely 0.019 units.

Table S1. General characteristics for the AKRIBEA sub-cohort.

	[ALL] *N=11159*	female *N=4544*	male *N=6615*	N
Sample type:				11159
Only serum	4702 (42.14%)	2262 (49.78%)	2440 (36.89%)	
Serum and Urine	6457 (57.86%)	2282 (50.22%)	4175 (63.11%)	
Age (years)	44.11±9.46	44.81±9.18	43.63±9.61	11159
Ethnicity:				10968
Asian	8 (0.07%)	2 (0.05%)	6 (0.09%)	
Black	16 (0.15%)	6 (0.14%)	10 (0.15%)	
Caucasian	8042 (73.32%)	3326 (74.96%)	4716 (72.21%)	
Hispanic	2885 (26.30%)	1099 (24.77%)	1786 (27.35%)	
Maghrebi	17 (0.15%)	4 (0.09%)	13 (0.20%)	
Weight (kg)	74.46±14.50	65.13±12.26	80.87±12.26	11159
Height (cm)	171.62±9.08	163.78±6.00	177.01±6.55	11159
BMI (kg/m²)	25.18±4.02	24.27±4.34	25.80±3.66	11159
Smoker	2084 (18.74%)	813 (17.96%)	1271 (19.28%)	11119
Drink alcohol:				11073
Never	1712 (15.46%)	990 (21.95%)	722 (11.00%)	
Social drinker	8442 (76.24%)	3339 (74.04%)	5103 (77.75%)	
Only during meals	633 (5.72%)	158 (3.50%)	475 (7.24%)	
Daily intake of alcohol	286 (2.58%)	23 (0.51%)	263 (4.01%)	
Hypertension	599 (5.37%)	166 (3.65%)	433 (6.55%)	11159
Medicated for hypertension	485 (4.35%)	140 (3.08%)	345 (5.22%)	11159
Medicated for diabetes	25 (1.17%)	3 (0.27%)	22 (2.18%)	2131
Medicated for hypercholesterolemia	206 (9.73%)	53 (4.74%)	153 (15.32%)	2117
ALT (U/L)	21.25±12.27	16.31±9.73	24.66±12.68	10974
Basophils (10^9/L)	0.04±0.02	0.04±0.02	0.05±0.02	11151
Cholesterol (mg/dL)	196.53±34.77	195.02±34.65	197.56±34.82	10974
HDL cholesterol (mg/dL)	61.73±15.92	68.55±16.02	57.02±14.04	10973
LDL cholesterol (mg/dL)	115.69±30.98	110.60±30.31	119.40±30.95	10454
Non-HDL cholesterol (mg/dL)	134.96±35.58	126.76±33.59	140.62±35.81	10973
Mean corpuscular hemoglobin (g/dL)	33.41±1.01	33.04±0.99	33.66±0.95	11151
Mean corpuscular volume (fL)	90.35±4.19	90.75±4.63	90.08±3.84	11151
Creatinine (mg/dL)	0.87±0.16	0.75±0.11	0.96±0.12	10974
Eosinophils (10^9/L)	0.22±0.16	0.20±0.15	0.24±0.17	11151
Erythrocytes (10^9/L)	4.83±0.43	4.51±0.33	5.05±0.35	11151
ESR (mm/h)	7.67±4.98	10.17±5.74	5.95±3.46	10906
GGT (U/L)	20.67±20.20	15.16±12.39	24.47±23.42	10974
Glucose (mg/dL)	87.74±12.83	86.41±10.61	88.66±14.08	10976
Hematocrit (%)	43.54±3.41	40.85±2.67	45.38±2.52	11151
Hemoglobin (g/dL)	14.55±1.28	13.50±0.98	15.27±0.90	11151
Leukocytes (10^9/L)	6.67±1.71	6.59±1.71	6.72±1.70	11151
Lymphocytes (10^9/L)	2.32±0.68	2.25±0.65	2.37±0.70	11151
Monocytes (10^9/L)	0.61±0.21	0.56±0.16	0.65±0.24	11151
Neutrophils (10^9/L)	3.47±1.30	3.54±1.33	3.42±1.27	11151
Platelets (10^9/L)	243.02±53.44	255.63±56.01	234.37±49.80	11151
Mean platelet volume (fL)	8.53±0.72	8.60±0.74	8.49±0.70	11148
RDW (%)	13.16±0.89	13.29±1.03	13.08±0.77	11103
Triglycerides (mg/dL)	94.68±58.69	79.55±39.85	105.11±66.78	10974
Urate (mg/dL)	5.08±1.29	4.17±0.96	5.71±1.11	10973

Table S2. General characteristics for the OSARTEN sub-cohort.

	[ALL] *N=8142*	female *N=2964*	male *N=5178*	N
Sample type:				8142
Only serum	569 (6.99%)	222 (7.49%)	347 (6.70%)	
Serum and Urine	7573 (93.01%)	2742 (92.51%)	4831 (93.30%)	
Age (years)	43.22±9.19	44.22±8.79	42.65±9.37	8142
Weight (kg)	75.59±14.22	65.09±11.37	81.59±12.04	8142
Height (cm)	172.00±9.02	163.68±6.34	176.77±6.52	8142
BMI (kg/m²)	25.44±3.81	24.30±4.05	26.10±3.49	8142
Smoker	1641 (20.18%)	556 (18.79%)	1085 (20.97%)	8133
Drink alcohol:				8124
Never	1138 (14.01%)	628 (21.23%)	510 (9.87%)	
Social drinker	6062 (74.62%)	2166 (73.23%)	3896 (75.42%)	
Only during meals	728 (8.96%)	144 (4.87%)	584 (11.30%)	
Daily intake of alcohol	196 (2.41%)	20 (0.68%)	176 (3.41%)	
Hypertension	927 (11.39%)	243 (8.20%)	684 (13.21%)	8142
Medicated for hypertension	82 (3.59%)	34 (3.67%)	48 (3.53%)	2285
Medicated for diabetes	70 (3.06%)	9 (0.97%)	61 (4.50%)	2284
Medicated for hypercholesterolemia	67 (2.93%)	26 (2.80%)	41 (3.02%)	2286
ALT (U/L)	22.78±13.87	16.62±9.46	26.31±14.73	8141
Basophils (10⁹/L)	0.04±0.02	0.04±0.02	0.04±0.02	8142
Cholesterol (mg/dL)	193.54±34.23	191.97±34.00	194.44±34.34	8142
HDL cholesterol (mg/dL)	60.63±15.82	68.40±15.28	56.18±14.33	8142
LDL cholesterol (mg/dL)	112.73±30.65	107.36±30.07	116.01±30.54	7680
Non-HDL cholesterol (mg/dL)	132.92±35.79	123.57±33.93	138.26±35.73	8142
Mean corpuscular hemoglobin (g/dL)	33.90±0.87	33.49±0.82	34.14±0.81	8142
Mean corpuscular volume (fL)	90.15±4.27	90.83±4.41	89.76±4.13	8142
Creatinine (mg/dL)	0.87±0.15	0.73±0.10	0.94±0.12	8142
Eosinophils (10⁹/L)	0.23±0.17	0.21±0.16	0.24±0.17	8142
Erythrocytes (10⁹/L)	4.84±0.41	4.50±0.31	5.03±0.34	8142
ESR (mm/h)	7.98±5.49	10.95±6.75	6.29±3.66	8141
GGT (U/L)	22.56±20.83	15.92±14.94	26.36±22.69	8140
Glucose (mg/dL)	86.09±12.50	84.60±10.74	86.95±13.32	8142
Hematocrit (%)	43.54±3.13	40.81±2.44	45.10±2.30	8142
Hemoglobin (g/dL)	14.77±1.21	13.67±0.92	15.39±0.85	8142
Leukocytes (10⁹/L)	6.73±1.70	6.60±1.67	6.80±1.71	8142
Lymphocytes (10⁹/L)	2.34±0.67	2.25±0.64	2.40±0.68	8142
Monocytes (10⁹/L)	0.62±0.19	0.57±0.17	0.65±0.20	8142
Neutrophils (10⁹/L)	3.50±1.26	3.53±1.28	3.48±1.24	8142
Platelets (10⁹/L)	238.42±51.88	249.93±55.06	231.83±48.76	8142
Mean platelet volume (fL)	8.40±0.68	8.46±0.70	8.36±0.67	8141
RDW (%)	13.33±0.79	13.40±0.89	13.29±0.71	8142
Triglycerides (mg/dL)	98.18±60.47	79.23±38.06	109.02±67.81	8142
Urate (mg/dL)	5.14±1.27	4.15±0.94	5.70±1.07	8142

Table S3. General characteristics for the LIVER-BIBLE sub-cohort.

	[ALL] *N=1057*	female *N=165*	male *N=892*	N
Sample type: Only serum	1057 (100.00%)	165 (100.00%)	892 (100.00%)	1057
Age (years)	54.00±6.32	53.76±6.10	54.04±6.37	1057
BMI (kg/m²)	28.63±3.24	28.92±3.86	28.58±3.12	1057
Ethnicity:				1057
Asian	29 (2.74%)	9 (5.45%)	20 (2.24%)	
Caucasian	1012 (95.74%)	150 (90.91%)	862 (96.64%)	
North African	11 (1.04%)	3 (1.82%)	8 (0.90%)	
South American	5 (0.47%)	3 (1.82%)	2 (0.22%)	
Hypertension	897 (84.86%)	136 (82.42%)	761 (85.31%)	1057
Glucose (mg/dL)	97.02±16.10	96.32±21.99	97.15±14.77	1057
Cholesterol (mg/dL)	201.92±33.07	207.79±35.02	200.83±32.60	1057
HDL cholesterol (mg/dL)	45.25±10.20	52.20±11.11	43.97±9.49	1057
LDL cholesterol (mg/dL)	124.18±31.01	126.29±32.34	123.79±30.77	1057
Triglycerides (mg/dL)	162.41±84.49	146.48±62.41	165.36±87.68	1057
Smoker	93 (8.82%)	12 (7.27%)	81 (9.11%)	1054
Medicated for hypertension	329 (31.13%)	32 (19.39%)	297 (33.30%)	1057
Medicated for hypercholesterolemia	116 (10.97%)	20 (12.12%)	96 (10.76%)	1057
Medicated for diabetes	4 (0.38%)	1 (0.61%)	3 (0.34%)	1057
Lymphocytes (10^9/L)	1.95±0.54	2.11±0.55	1.92±0.53	1051
Monocytes (10^9/L)	0.53±0.14	0.49±0.14	0.53±0.14	1051
Eosinophils (10^9/L)	0.16±0.11	0.15±0.11	0.16±0.11	1051
Basophils (10^9/L)	0.04±0.02	0.04±0.02	0.04±0.02	1051
Hemoglobin (g/dL)	14.82±1.07	13.42±0.87	15.07±0.88	1051
Hematocrit (%)	42.69±2.85	39.47±2.52	43.28±2.49	1051
Mean corpuscular volume (fL)	85.65±3.88	85.83±3.63	85.61±3.92	1051
Mean corpuscular hemoglobin (pg)	29.72±1.66	29.21±1.56	29.81±1.66	1051
RDW (%)	12.99±0.83	13.18±0.95	12.95±0.80	1051
Platelets (10^9/L)	232.47±50.04	253.51±55.28	228.60±48.07	1051
Creatinine (mg/dL)	0.99±0.16	0.80±0.15	1.02±0.14	1056
ALT (U/L)	29.96±13.53	22.25±9.32	31.38±13.72	1057
GGT (U/L)	27.50±18.46	17.64±9.37	29.32±19.14	1057
AST (U/L)	24.07±7.74	20.86±6.21	24.66±7.85	1057
Ferritin (ng/mL)	110.76±110.04	70.05±90.92	118.29±111.65	1057
Systolic blood pressure (mmHG)	137.14±12.89	137.60±12.57	137.05±12.96	1057
Diastolic blood pressure (mmHG)	85.89±8.02	84.55±8.80	86.14±7.84	1057
Waist circumference (cm)	102.95±9.05	98.94±9.15	103.70±8.83	1056
eGFR (CKD-EPI) (mL/min/1.73m²)	83.69±12.97	84.64±15.89	83.52±12.36	1057
Total protein (g/dL)	7.07±0.34	7.02±0.37	7.08±0.33	989
PCSK9 (ng/mL)	295.59±94.17	325.73±110.42	291.42±91.11	379
Hypercholesterolemia	507 (47.97%)	83 (50.30%)	424 (47.53%)	1057
HbA1c (mmol/mol)	35.79±4.82	37.25±7.07	35.52±4.23	1057
Insulin (µIU/mL)	14.79±9.08	14.01±10.01	14.93±8.90	1057
HOMA-IR	3.56±2.40	3.41±2.97	3.59±2.28	1057
Urine ACR (mg/g)	11.09±25.69	14.88±46.72	10.40±19.56	982
Albuminuria	48 (4.89%)	6 (3.97%)	42 (5.05%)	982
Hyperglycemia	77 (7.28%)	21 (12.73%)	56 (6.28%)	1057
Type 2 diabetes	15 (1.42%)	5 (3.03%)	10 (1.12%)	1057
RBC (10^6/µL)	5.00±0.39	4.61±0.33	5.07±0.36	1051
WBC (10^9/L)	6.27±1.40	6.47±1.44	6.24±1.39	1051

Table S4. General characteristics for the METS+ sub-cohort.

	[ALL]<br>*N=147*	female<br>*N=49*	male<br>*N=98*	N
Sample type: Only serum	147 (100.00%)	49 (100.00%)	98 (100.00%)	147
Age (years)	66.88±13.00	71.29±10.83	64.67±13.47	147
Weight (kg)	90.16±23.30	82.15±16.96	94.17±25.02	147
Height (cm)	165.70±9.91	156.33±7.31	170.38±7.40	147
BMI (kg/m²)	32.76±7.34	33.63±6.35	32.33±7.79	147
Waist circumference (cm)	110.25±14.08	108.76±13.41	111.02±14.44	129
Diabetes: yes	147 (100.00%)	49 (100.00%)	98 (100.00%)	147
Obesity	52 (89.66%)	18 (90.00%)	34 (89.47%)	58
Dyslipidemia	41 (70.69%)	13 (65.00%)	28 (73.68%)	58
Hypertension	110 (74.83%)	37 (75.51%)	73 (74.49%)	147
Glucose (mg/dL)	141.82±53.42	145.37±60.97	140.03±49.42	146
Cholesterol (mg/dL)	136.68±67.17	131.88±73.72	139.08±63.90	147
HDL cholesterol (mg/dL)	72.07±35.76	81.17±38.02	67.67±34.07	89
LDL cholesterol (mg/dL)	117.73±59.09	136.48±62.20	108.67±55.81	89
Triglycerides (mg/dL)	144.61±72.74	161.31±82.69	136.26±66.10	147
Systolic blood pressure (mmHG)	142.68±16.34	140.92±17.05	143.41±16.29	41
Diastolic blood pressure (mmHG)	79.83±10.28	74.92±11.64	81.86±9.12	41

Table S5. General characteristics for the AGEPORTUGAL sub-cohort.

	[ALL] *N=157*	female *N=99*	male *N=58*	N
Sample type:				157
Only serum	76 (48.41%)	56 (56.57%)	20 (34.48%)	
Serum and Urine	81 (51.59%)	43 (43.43%)	38 (65.52%)	
Age (years)	82.48±7.66	83.42±7.07	80.88±8.39	157
Weight (kg)	65.24±13.04	63.26±11.38	68.62±14.97	157
Height (cm)	155.65±8.50	151.39±6.35	162.91±6.59	157
BMI (kg/m²)	26.88±4.67	27.57±4.56	25.72±4.66	157
Smoker	7 (4.49%)	1 (1.02%)	6 (10.34%)	156
Diabetes	45 (31.91%)	34 (37.78%)	11 (21.57%)	141
Medicated for diabetes	36 (23.84%)	29 (30.21%)	7 (12.73%)	151
Dyslipidemia	71 (48.97%)	52 (56.52%)	19 (35.85%)	145
Medicated for hypercholesterolemia	64 (42.38%)	47 (48.96%)	17 (30.91%)	151
Hypertension	114 (76.00%)	71 (75.53%)	43 (76.79%)	150
Medicated for hypertension	123 (81.46%)	80 (83.33%)	43 (78.18%)	151
Glucose (mg/dL)	100.99±28.29	102.20±30.15	98.97±25.01	152
Cholesterol (mg/dL)	164.62±47.91	171.84±45.35	152.60±50.03	152
HDL cholesterol (mg/dL)	56.35±13.21	57.67±14.59	54.14±10.27	152
LDL cholesterol (mg/dL)	85.60±41.09	89.23±39.18	79.54±43.76	152
Triglycerides (mg/dL)	113.41±53.37	124.70±57.05	94.58±40.50	152
Systolic blood pressure (mmHG)	126.32±20.72	125.66±20.98	127.35±20.46	146
Diastolic blood pressure (mmHG)	70.13±11.18	69.30±11.28	71.42±10.99	146
Drink alcohol:				154
Never	133 (86.36%)	90 (93.75%)	43 (74.14%)	
Social drinker	20 (12.99%)	6 (6.25%)	14 (24.14%)	
Daily intake of alcohol	1 (0.65%)	0 (0.00%)	1 (1.72%)	
Atrial fibrillation	15 (10.34%)	11 (12.22%)	4 (7.27%)	145
Atherosclerosis	7 (4.96%)	4 (4.49%)	3 (5.77%)	141
Acute myocardial infarction	5 (3.55%)	2 (2.25%)	3 (5.77%)	141
Chronic congestive heart failure	26 (18.44%)	19 (21.35%)	7 (13.46%)	141
Angina pectoris	7 (4.96%)	4 (4.49%)	3 (5.77%)	141
Stroke	19 (13.48%)	11 (12.36%)	8 (15.38%)	141
Cardiac arrhythmias: no	143 (100.00%)	91 (100.00%)	52 (100.00%)	143
Peripheral vascular disease	10 (6.99%)	7 (7.69%)	3 (5.77%)	143
Dementia	29 (20.14%)	22 (23.91%)	7 (13.46%)	144
ACE-R total	49.27±19.24	48.46±18.86	50.71±20.01	144
Mini Mental	17.91±5.60	17.72±5.18	18.24±6.28	147
FAB total	7.82±3.44	7.47±3.33	8.39±3.58	149
EUROHIS total	58.22±14.81	57.49±15.46	59.38±13.77	151
Former smoker	24 (16.55%)	3 (3.16%)	21 (42.00%)	145

Table S6. General characteristics for the Validation cohorts.

				BIOPERSMED *N=279*	BPH *N=324*	TÜBINGEN *N=58*
**Gender: female**				144 (51.6%)	0 (0.00%)	29 (50.0%)
**Age, median (IQR)**				57 (51.0-63.0)	71 (65.0-77.0)	56.50 (52.2-60.8)
**Medication**						
Antidiabetic				50 (17.9%)	57 (18.6%)	n.a.*
Lipid-lowering				42 (15.1%)	99 (30.6%)	n.a.*
Antihypertensive				98 (35.1%)	124 (38.3%)	58 (100%)
**MetS profile**						
diabetes	obesity	dyslipidemia	hypertension			
	(0 - no; 1 - yes)					
0	0	0	0	64 (22.9%)	81 (25.0%)	0 (0.00%)
0	0	0	1	53 (19.0%)	43 (13.3%)	1 (1.72%)
0	0	1	0	27 (9.68%)	43 (13.3%)	0 (0.00%)
0	0	1	1	27 (9.68%)	39 (12.0%)	3 (5.17%)
0	1	0	0	5 (1.79%)	19 (5.86%)	0 (0.00%)
0	1	0	1	16 (5.73%)	7 (2.16%)	1 (1.72%)
0	1	1	0	10 (3.58%)	13 (4.01%)	0 (0.00%)
0	1	1	1	27 (9.68%)	9 (2.78%)	4 (6.90%)
1	0	0	0	24 (8.60%)	9 (2.78%)	0 (0.00%)
1	0	0	1	10 (3.58%)	15 (4.63%)	4 (6.90%)
1	0	1	0	6 (2.15%)	13 (4.01%)	0 (0.00%)
1	0	1	1	4 (1.43%)	14 (4.32%)	17 (29.3%)
1	1	0	0	2 (0.72%)	2 (0.62%)	0 (0.00%)
1	1	0	1	1 (0.36%)	4 (1.23%)	5 (8.62%)
1	1	1	0	1 (0.36%)	4 (1.23%)	0 (0.00%)
1	1	1	1	2 (0.72%)	9 (2.78%)	23 (39.7%)
**Has MetS WHO?**				8 (2.87%)	31 (9.57%)	45 (77.6%)

*n.a.: not available

Table S7. Metabolic syndrome risk factors.

Conditions* (RF_1, RF_2, RF_3, RF_4)			
**RF_1 (Pre)Diabetes**	**RF_2 Obesity**	**RF_3 Dyslipidemia**	**RF_4 Hypertension**
Fasting plasma glucose > 110 mg/dL<br><br>Previously diagnosed type 2 diabetes, impaired fasting glucose, impaired glucose tolerance or insulin resistance	BMI > 30 kg/m^2	Triglycerides > 150 mg/dL<br><br>HDL cholesterol < 34.75 mg/dL in men or < 38.61 in women<br><br>Previously diagnosed hypercholesterolemia, hyperlipidemia or hypertriglyceridemia<br><br>Taking medication for dyslipidemia	Blood pressure ≥ 140/90 mmHg<br><br>Previously diagnosed hypertension<br><br>Taking medication for hypertension

***RF: risk factor.**

Table S8. List of metabolites and lipoproteins that are quantified in serum samples with Bruker IVDR software. Sometimes lipoproteins are referred to by their short code, which is also indicated.

Bruker's Software	Group	Variable	Code	Unit
B.I.LISA	Lipoproteins: Apo-A1	Apo-A1	TPA1	mg/dL
B.I.LISA	Lipoproteins: Apo-A1	HDL Apo-A1	HDA1	mg/dL
B.I.LISA	Lipoproteins: Apo-A1	HDL-1 Apo-A1	H1A1	mg/dL
B.I.LISA	Lipoproteins: Apo-A1	HDL-2 Apo-A1	H2A1	mg/dL
B.I.LISA	Lipoproteins: Apo-A1	HDL-3 Apo-A1	H3A1	mg/dL
B.I.LISA	Lipoproteins: Apo-A1	HDL-4 Apo-A1	H4A1	mg/dL
B.I.LISA	Lipoproteins: Apo-A2	Apo-A2	TPA2	mg/dL
B.I.LISA	Lipoproteins: Apo-A2	HDL Apo-A2	HDA2	mg/dL
B.I.LISA	Lipoproteins: Apo-A2	HDL-1 Apo-A2	H1A2	mg/dL
B.I.LISA	Lipoproteins: Apo-A2	HDL-2 Apo-A2	H2A2	mg/dL
B.I.LISA	Lipoproteins: Apo-A2	HDL-3 Apo-A2	H3A2	mg/dL
B.I.LISA	Lipoproteins: Apo-A2	HDL-4 Apo-A2	H4A2	mg/dL
B.I.LISA	Lipoproteins: Apo-B	Apo-B	TPAB	mg/dL
B.I.LISA	Lipoproteins: Apo-B	LDL Apo-B	LDAB	mg/dL
B.I.LISA	Lipoproteins: Apo-B	LDL-1 Apo-B	L1AB	mg/dL
B.I.LISA	Lipoproteins: Apo-B	LDL-2 Apo-B	L2AB	mg/dL
B.I.LISA	Lipoproteins: Apo-B	LDL-3 Apo-B	L3AB	mg/dL
B.I.LISA	Lipoproteins: Apo-B	LDL-4 Apo-B	L4AB	mg/dL
B.I.LISA	Lipoproteins: Apo-B	LDL-5 Apo-B	L5AB	mg/dL
B.I.LISA	Lipoproteins: Apo-B	LDL-6 Apo-B	L6AB	mg/dL
B.I.LISA	Lipoproteins: Apo-B	IDL Apo-B	IDAB	mg/dL
B.I.LISA	Lipoproteins: Apo-B	VLDL Apo-B	VLAB	mg/dL
B.I.LISA	Lipoproteins: Phospholipids	HDL Phospholipids	HDPL	mg/dL
B.I.LISA	Lipoproteins: Phospholipids	HDL-1 Phospholipids	H1PL	mg/dL
B.I.LISA	Lipoproteins: Phospholipids	HDL-2 Phospholipids	H2PL	mg/dL
B.I.LISA	Lipoproteins: Phospholipids	HDL-3 Phospholipids	H3PL	mg/dL
B.I.LISA	Lipoproteins: Phospholipids	HDL-4 Phospholipids	H4PL	mg/dL
B.I.LISA	Lipoproteins: Phospholipids	LDL Phospholipids	LDPL	mg/dL
B.I.LISA	Lipoproteins: Phospholipids	LDL-1 Phospholipids	L1PL	mg/dL
B.I.LISA	Lipoproteins: Phospholipids	LDL-2 Phospholipids	L2PL	mg/dL
B.I.LISA	Lipoproteins: Phospholipids	LDL-3 Phospholipids	L3PL	mg/dL
B.I.LISA	Lipoproteins: Phospholipids	LDL-4 Phospholipids	L4PL	mg/dL
B.I.LISA	Lipoproteins: Phospholipids	LDL-5 Phospholipids	L5PL	mg/dL
B.I.LISA	Lipoproteins: Phospholipids	LDL-6 Phospholipids	L6PL	mg/dL
B.I.LISA	Lipoproteins: Phospholipids	IDL Phospholipids	IDPL	mg/dL
B.I.LISA	Lipoproteins: Phospholipids	VLDL Phospholipids	VLPL	mg/dL
B.I.LISA	Lipoproteins: Phospholipids	VLDL-1 Phospholipids	V1PL	mg/dL
B.I.LISA	Lipoproteins: Phospholipids	VLDL-2 Phospholipids	V2PL	mg/dL
B.I.LISA	Lipoproteins: Phospholipids	VLDL-3 Phospholipids	V3PL	mg/dL
B.I.LISA	Lipoproteins: Phospholipids	VLDL-4 Phospholipids	V4PL	mg/dL
B.I.LISA	Lipoproteins: Phospholipids	VLDL-5 Phospholipids	V5PL	mg/dL
B.I.LISA	Lipoproteins: Calculated parameters	Apo-B/Apo-A1	ABA1	-
B.I.LISA	Lipoproteins: Calculated parameters	LDL-Chol/HDL-Chol	LDHD	-
B.I.LISA	Lipoproteins: Cholesterol	Total Cholesterol	TPCH	mg/dL
B.I.LISA	Lipoproteins: Cholesterol	HDL Cholesterol	HDCH	mg/dL
B.I.LISA	Lipoproteins: Cholesterol	HDL Cholesterol	HDCH	mg/dL
B.I.LISA	Lipoproteins: Cholesterol	HDL-1 Cholesterol	H1CH	mg/dL
B.I.LISA	Lipoproteins: Cholesterol	HDL-2 Cholesterol	H2CH	mg/dL
B.I.LISA	Lipoproteins: Cholesterol	HDL-3 Cholesterol	H3CH	mg/dL
B.I.LISA	Lipoproteins: Cholesterol	HDL-4 Cholesterol	H4CH	mg/dL
B.I.LISA	Lipoproteins: Cholesterol	LDL Cholesterol	LDCH	mg/dL
B.I.LISA	Lipoproteins: Cholesterol	LDL Cholesterol	LDCH	mg/dL
B.I.LISA	Lipoproteins: Cholesterol	LDL-1 Cholesterol	L1CH	mg/dL
B.I.LISA	Lipoproteins: Cholesterol	LDL-2 Cholesterol	L2CH	mg/dL
B.I.LISA	Lipoproteins: Cholesterol	LDL-3 Cholesterol	L3CH	mg/dL
B.I.LISA	Lipoproteins: Cholesterol	LDL-4 Cholesterol	L4CH	mg/dL
B.I.LISA	Lipoproteins: Cholesterol	LDL-5 Cholesterol	L5CH	mg/dL
B.I.LISA	Lipoproteins: Cholesterol	LDL-6 Cholesterol	L6CH	mg/dL
B.I.LISA	Lipoproteins: Cholesterol	IDL Cholesterol	IDCH	mg/dL
B.I.LISA	Lipoproteins: Cholesterol	VLDL Cholesterol	VLCH	mg/dL
B.I.LISA	Lipoproteins: Cholesterol	VLDL-1 Cholesterol	V1CH	mg/dL
B.I.LISA	Lipoproteins: Cholesterol	VLDL-2 Cholesterol	V2CH	mg/dL
B.I.LISA	Lipoproteins: Cholesterol	VLDL-3 Cholesterol	V3CH	mg/dL
B.I.LISA	Lipoproteins: Cholesterol	VLDL-4 Cholesterol	V4CH	mg/dL
B.I.LISA	Lipoproteins: Cholesterol	VLDL-5 Cholesterol	V5CH	mg/dL
B.I.LISA	Lipoproteins: Free Cholesterol	HDL Free Cholesterol	HDFC	mg/dL
B.I.LISA	Lipoproteins: Free Cholesterol	HDL-1 Free Cholesterol	H1FC	mg/dL
B.I.LISA	Lipoproteins: Free Cholesterol	HDL-2 Free Cholesterol	H2FC	mg/dL
B.I.LISA	Lipoproteins: Free Cholesterol	HDL-3 Free Cholesterol	H3FC	mg/dL
B.I.LISA	Lipoproteins: Free Cholesterol	HDL-4 Free Cholesterol	H4FC	mg/dL

B.I.LISA	Lipoproteins: Free Cholesterol	LDL Free Cholesterol	LDFC	mg/dL
B.I.LISA	Lipoproteins: Free Cholesterol	LDL-1 Free Cholesterol	L1FC	mg/dL
B.I.LISA	Lipoproteins: Free Cholesterol	LDL-2 Free Cholesterol	L2FC	mg/dL
B.I.LISA	Lipoproteins: Free Cholesterol	LDL-3 Free Cholesterol	L3FC	mg/dL
B.I.LISA	Lipoproteins: Free Cholesterol	LDL-4 Free Cholesterol	L4FC	mg/dL
B.I.LISA	Lipoproteins: Free Cholesterol	LDL-5 Free Cholesterol	L5FC	mg/dL
B.I.LISA	Lipoproteins: Free Cholesterol	LDL-6 Free Cholesterol	L6FC	mg/dL
B.I.LISA	Lipoproteins: Free Cholesterol	IDL Free Cholesterol	IDFC	mg/dL
B.I.LISA	Lipoproteins: Free Cholesterol	VLDL Free Cholesterol	VLFC	mg/dL
B.I.LISA	Lipoproteins: Free Cholesterol	VLDL-1 Free Cholesterol	V1FC	mg/dL
B.I.LISA	Lipoproteins: Free Cholesterol	VLDL-2 Free Cholesterol	V2FC	mg/dL
B.I.LISA	Lipoproteins: Free Cholesterol	VLDL-3 Free Cholesterol	V3FC	mg/dL
B.I.LISA	Lipoproteins: Free Cholesterol	VLDL-4 Free Cholesterol	V4FC	mg/dL
B.I.LISA	Lipoproteins: Free Cholesterol	VLDL-5 Free Cholesterol	V5FC	mg/dL
B.I.LISA	Lipoproteins: Triglycerides	Triglycerides	TPTG	mg/dL
B.I.LISA	Lipoproteins: Triglycerides	HDL Triglycerides	HDTG	mg/dL
B.I.LISA	Lipoproteins: Triglycerides	HDL-1 Triglycerides	H1TG	mg/dL
B.I.LISA	Lipoproteins: Triglycerides	HDL-2 Triglycerides	H2TG	mg/dL
B.I.LISA	Lipoproteins: Triglycerides	HDL-3 Triglycerides	H3TG	mg/dL
B.I.LISA	Lipoproteins: Triglycerides	HDL-4 Triglycerides	H4TG	mg/dL
B.I.LISA	Lipoproteins: Triglycerides	LDL Triglycerides	LDTG	mg/dL
B.I.LISA	Lipoproteins: Triglycerides	LDL-1 Triglycerides	L1TG	mg/dL
B.I.LISA	Lipoproteins: Triglycerides	LDL-2 Triglycerides	L2TG	mg/dL
B.I.LISA	Lipoproteins: Triglycerides	LDL-3 Triglycerides	L3TG	mg/dL
B.I.LISA	Lipoproteins: Triglycerides	LDL-4 Triglycerides	L4TG	mg/dL
B.I.LISA	Lipoproteins: Triglycerides	LDL-5 Triglycerides	L5TG	mg/dL
B.I.LISA	Lipoproteins: Triglycerides	LDL-6 Triglycerides	L6TG	mg/dL
B.I.LISA	Lipoproteins: Triglycerides	IDL Triglycerides	IDTG	mg/dL
B.I.LISA	Lipoproteins: Triglycerides	VLDL Triglycerides	VLTG	mg/dL
B.I.LISA	Lipoproteins: Triglycerides	VLDL-1 Triglycerides	V1TG	mg/dL
B.I.LISA	Lipoproteins: Triglycerides	VLDL-2 Triglycerides	V2TG	mg/dL
B.I.LISA	Lipoproteins: Triglycerides	VLDL-3 Triglycerides	V3TG	mg/dL
B.I.LISA	Lipoproteins: Triglycerides	VLDL-4 Triglycerides	V4TG	mg/dL
B.I.LISA	Lipoproteins: Triglycerides	VLDL-5 Triglycerides	V5TG	mg/dL
B.I.Quant-PS	Metabolites	2-Aminobutyric acid	-	mmol/L
B.I.Quant-PS	Metabolites	2-Hydroxybutyric acid	-	mmol/L
B.I.Quant-PS	Metabolites	2-Oxoglutaric acid	-	mmol/L
B.I.Quant-PS	Metabolites	3-Hydroxybutyric acid	-	mmol/L
B.I.Quant-PS	Metabolites	Acetic acid	-	mmol/L
B.I.Quant-PS	Metabolites	Acetoacetic acid	-	mmol/L
B.I.Quant-PS	Metabolites	Acetone	-	mmol/L
B.I.Quant-PS	Metabolites	Alanine	-	mmol/L
B.I.Quant-PS	Metabolites	Asparagine	-	mmol/L
B.I.Quant-PS	Metabolites	Choline	-	mmol/L
B.I.Quant-PS	Metabolites	Citric acid	-	mmol/L
B.I.Quant-PS	Metabolites	Creatine	-	mmol/L
B.I.Quant-PS	Metabolites	Creatinine	-	mmol/L
B.I.Quant-PS	Metabolites	D-Galactose	-	mmol/L
B.I.Quant-PS	Metabolites	Dimethylsulfone	-	mmol/L
B.I.Quant-PS	Metabolites	Ethanol	-	mmol/L
B.I.Quant-PS	Metabolites	Formic acid	-	mmol/L
B.I.Quant-PS	Metabolites	Glucose	-	mmol/L
B.I.Quant-PS	Metabolites	Glutamic acid	-	mmol/L
B.I.Quant-PS	Metabolites	Glutamine	-	mmol/L
B.I.Quant-PS	Metabolites	Glycerol	-	mmol/L
B.I.Quant-PS	Metabolites	Glycine	-	mmol/L
B.I.Quant-PS	Metabolites	Histidine	-	mmol/L
B.I.Quant-PS	Metabolites	Isoleucine	-	mmol/L
B.I.Quant-PS	Metabolites	Lactic acid	-	mmol/L
B.I.Quant-PS	Metabolites	Leucine	-	mmol/L
B.I.Quant-PS	Metabolites	Lysine	-	mmol/L
B.I.Quant-PS	Metabolites	Methionine	-	mmol/L
B.I.Quant-PS	Metabolites	N,N-Dimethylglycine	-	mmol/L
B.I.Quant-PS	Metabolites	Ornithine	-	mmol/L
B.I.Quant-PS	Metabolites	Phenylalanine	-	mmol/L
B.I.Quant-PS	Metabolites	Proline	-	mmol/L
B.I.Quant-PS	Metabolites	Pyruvic acid	-	mmol/L
B.I.Quant-PS	Metabolites	Sarcosine	-	mmol/L
B.I.Quant-PS	Metabolites	Succinic acid	-	mmol/L
B.I.Quant-PS	Metabolites	Threonine	-	mmol/L
B.I.Quant-PS	Metabolites	Trimethylamine-N-oxide	-	mmol/L
B.I.Quant-PS	Metabolites	Tyrosine	-	mmol/L
B.I.Quant-PS	Metabolites	Valine	-	mmol/L

Table S9. Performance metrics of generated *metabo/lipo-serum* predictive models for MetS. AUROC (Area Under Receiver Operating Characteristic curve) also indicates 95% interval in brackets. Repeated cross-validations indicates estimated *P*-value (*p*) from 100 random permutations, and confidence intervals for sensitivity and specificity.

	AUROC	Sensitivity	Specificity
**Train set (80%)**	0.932 [0.913-0.950]	0.858	0.909
**Test set (20%)**	0.943 [0.914-0.973]	0.882	0.908
**Repeated Cross-validation (100%, 10x5-fold)**	0.935 [0.903-0.960] (p <0.01)	0.869 [0.805-0.927] (p <0.01)	0.913 [0.865-0.951] (p <0.01)
**Final model (100%)**	0.936 [0.920-0.952]	0.861	0.915

Table S10. Loading values for the predictive component of the *metabo/lipo-serum* O-PLS-DA model.

Variable	Loading value	Ranking
Glucose	0.561	1
Ala-Tyr	0.2959	2
HDLs 1-3 noTGs	-0.2618	3
VLDLs	0.2553	4
LDLs 4-5	0.2446	5
Formic acid	0.2439	6
LDLs 1-3	-0.2419	7
Proline	0.214	8
LDLs 6	0.2082	9
Citric acid	0.1893	10
Iso-Leu-Val	0.1852	11
HDLs 1-3 TGs	0.1542	12
Creatinine	0.1499	13
HDLs 4 noTGs	-0.1396	14
Phenylalanine	0.1174	15
Glycine	-0.1111	16
Glutamic acid	0.1065	17
Creatine	0.0826	18
Lact-Pyru.acids	0.0788	19
Methionine	-0.0752	20
Sarcosine	0.064	21
Trimethylamine-N-oxide	0.0313	22

Table S11. Performance metrics of limited *metabo/lipo-serum* predictive models: excluding Glucose, HDLs and LDLs. AUROC (Area Under Receiver Operating Characteristic curve) also indicates 95% interval in brackets. Repeated cross-validations indicates estimated *P*-value (*p*) from 100 random permutations, and confidence intervals for sensitivity and specificity.

	AUROC	Sensitivity	Specificity
**Train set (80%)**	0.890 [0.872-0.908]	0.868	0.757
**Test set (20%)**	0.901 [0.873-0.930]	0.871	0.754
**Repeated Cross-validation (100%, 10x5-fold)**	0.891 [0.857-0.920] (p <0.01)	0.830 [0.706-0.927] (p <0.01)	0.812 [0.725-0.935] (p <0.01)
**Final model (100%)**	0.895 [0.880-0.910]	0.863	0.769

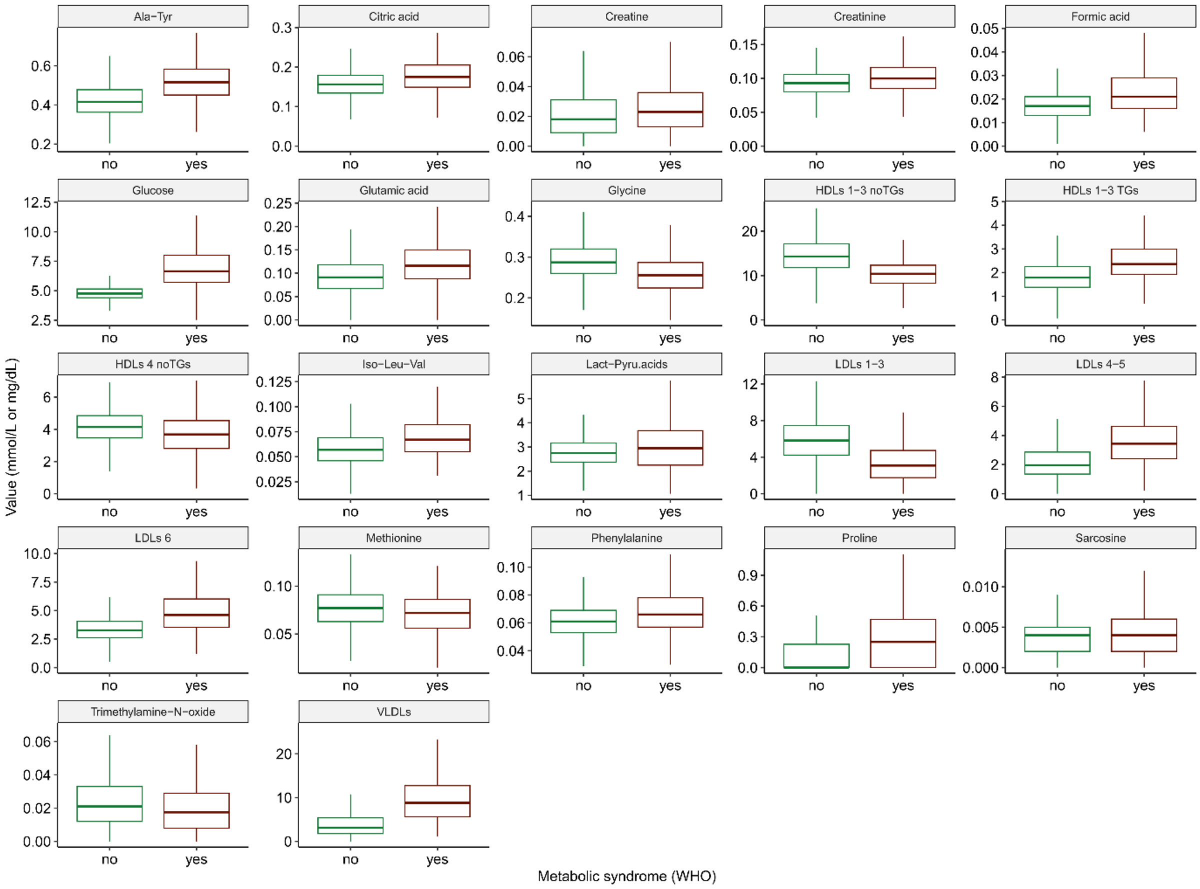

Figure S1. Comparison of raw quantifications (MetS_WHO vs. other). Representation in boxplot format of the distribution of quantification values of selected variables for the *metabo/lipo-serum* predictive model, segregated based on whether they belong to individuals classified with metabolic syndrome according to WHO criteria, compared to the remaining individuals. Quantification units are mmol/L for metabolites and mg/dL for lipoproteins. Outliers have been omitted in the boxplots to enhance visualization.

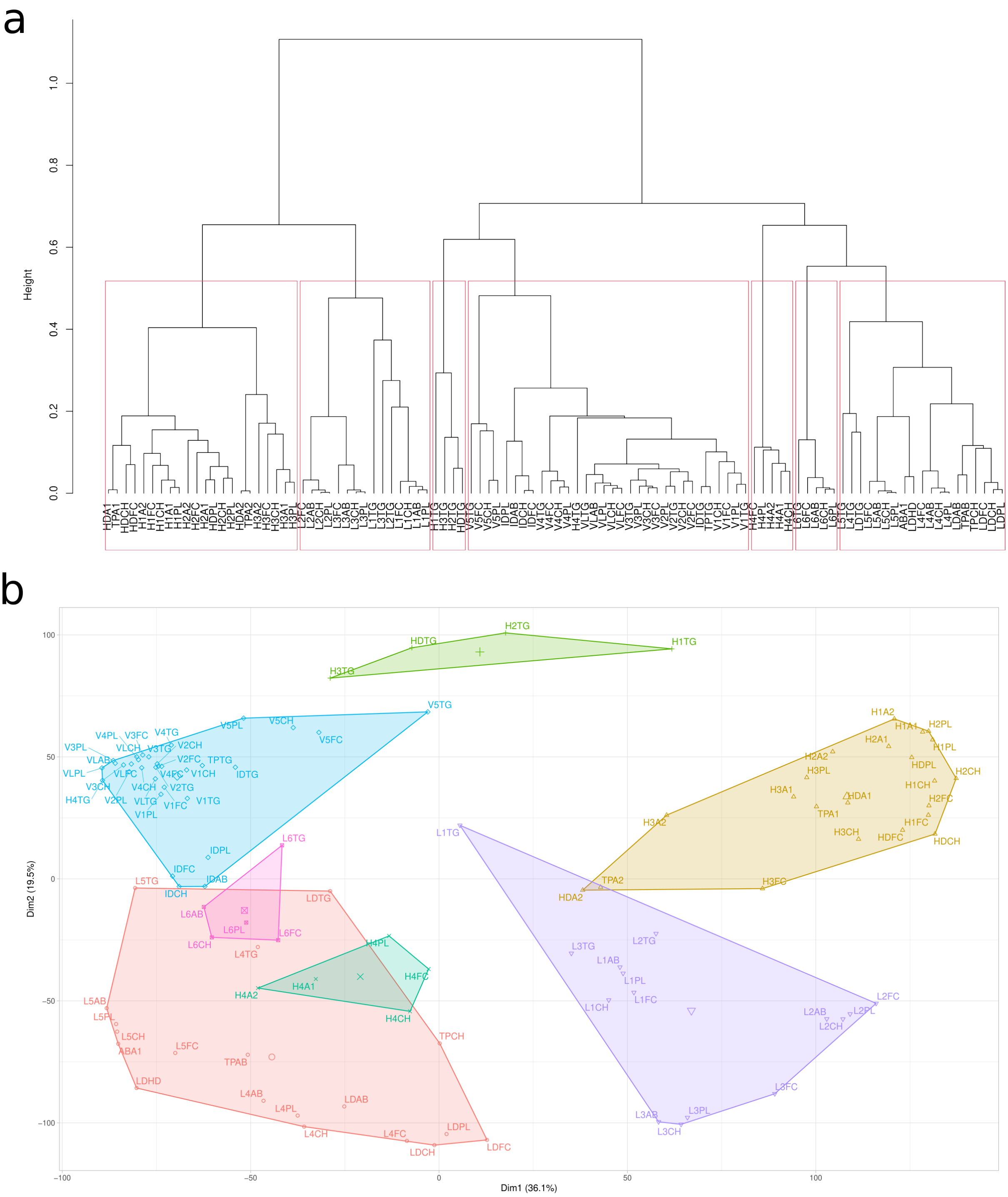

Figure S2. Clustering of serum lipoproteins. Panel a show a dendrogram generated from the hierarchical clustering analysis performed on serum lipoprotein values. The red rectangles represent the groups formed when the dendrogram tree is cut at a height of h=0.5. Those clusters are represented in panel b, which is a scores plot from a PCA of variables.

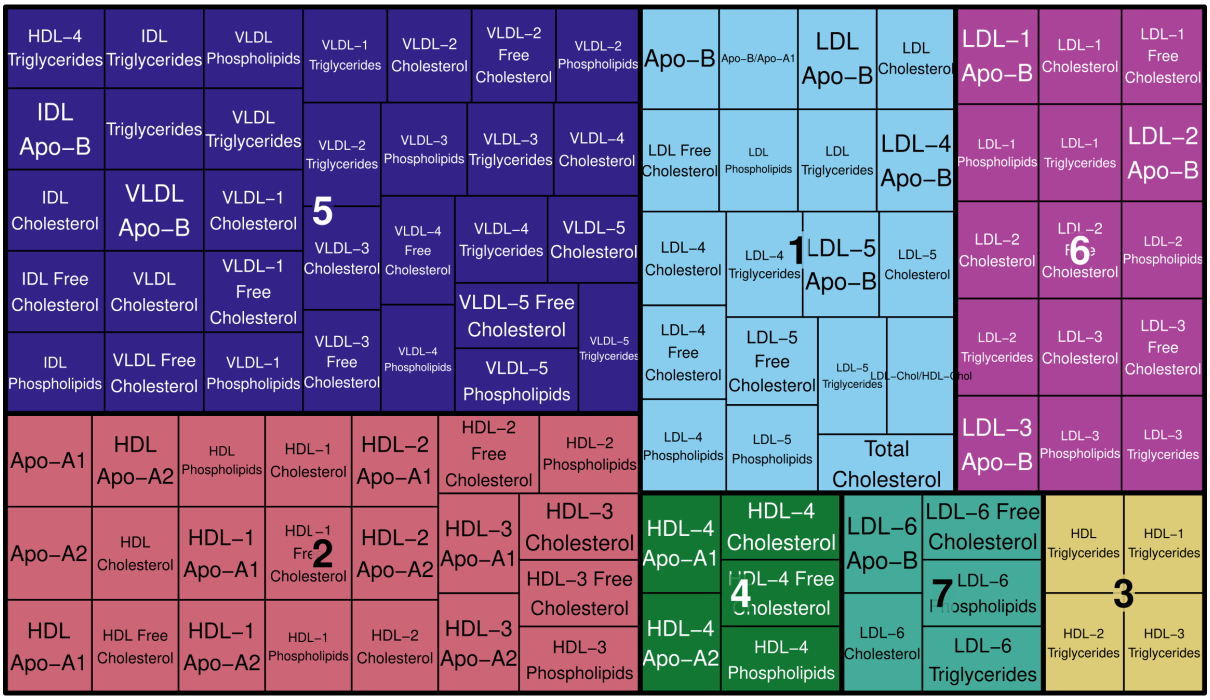

Figure S3. Mosaic plot with lipoprotein clusters and their components. Each cluster is represented by a color and a number from 1 to 7. The inner rectangles are the variables included in the cluster. For quick referencing, each cluster receives these short names: 1) LDLs 4-5; 2) HDLs 1-3 noTGs; 3) HDLs 1-3 TGs; 4) HDLs 4 noTGs; 5) VLDLs; 6) LDLs 1-3; and 7) LDLs 6.

a

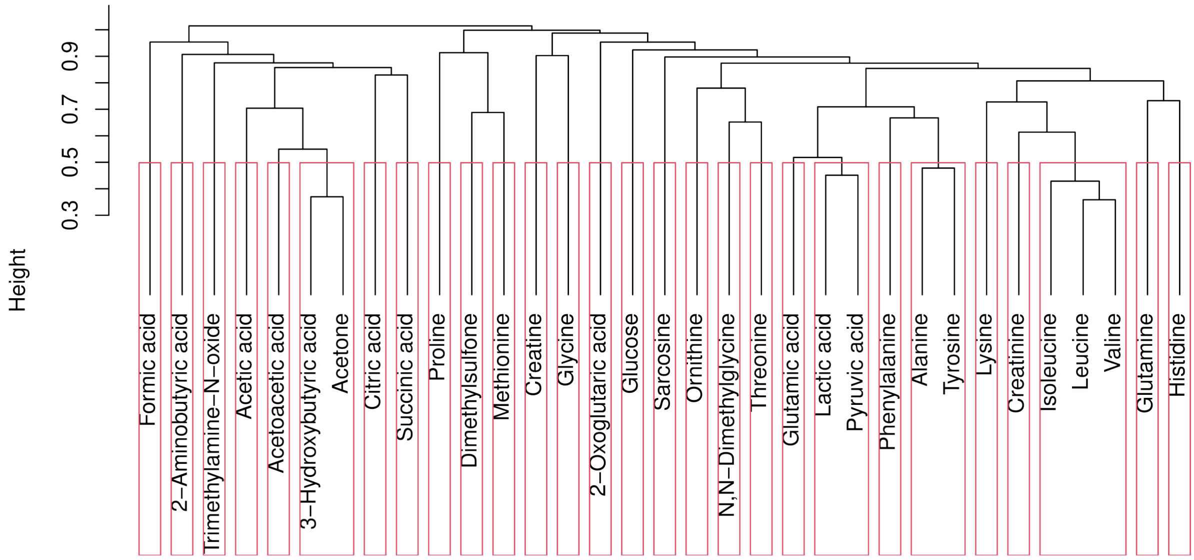

b

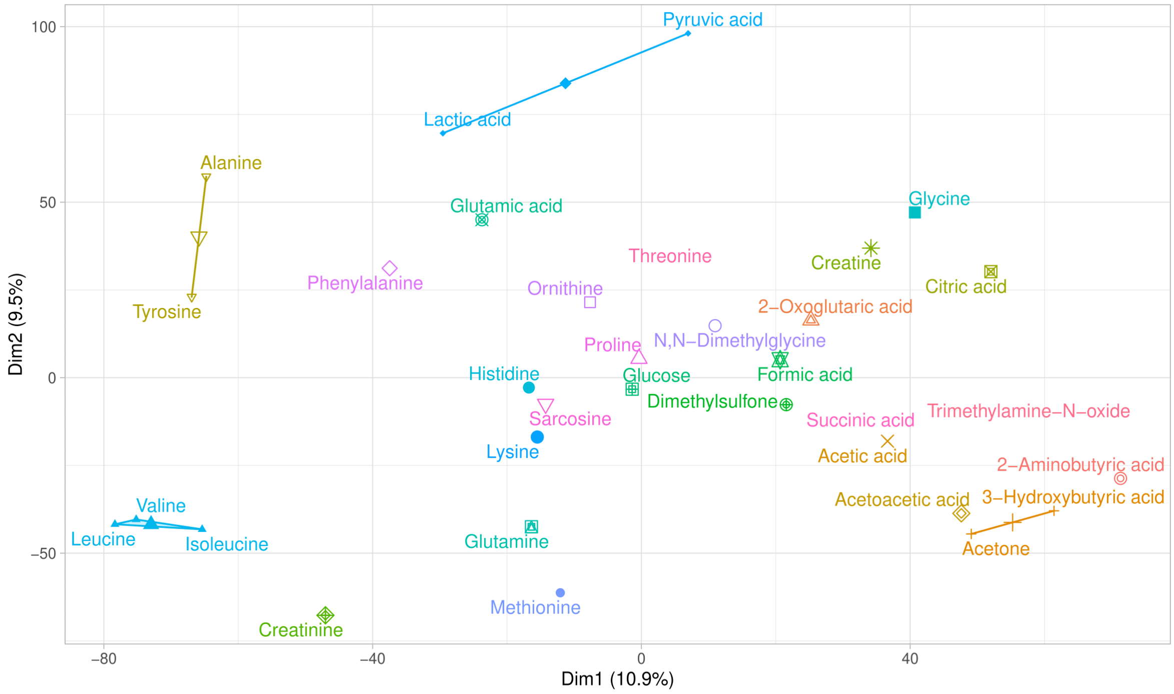

Figure S4. Clustering of serum metabolites. Panel a show a dendrogram generated from the hierarchical clustering analysis performed on serum metabolite values. The red rectangles represent the groups formed when the dendrogram tree is cut at a height of h=0.5. Those clusters are represented in panel b, which is a scores plot from a PCA of variables. For a quick reference, clusters with more than one component receive these names: Ala-Tyr (Alanine+Tyrosine); Iso-Leu-Val (Isoleucine+Leurine+Valine); Lact-Pyru.acids (Lactic acid+Pyruvic acid); and Aceto-3Hydroxybut (Acetone+3-Hydroxybutyric acid).

a

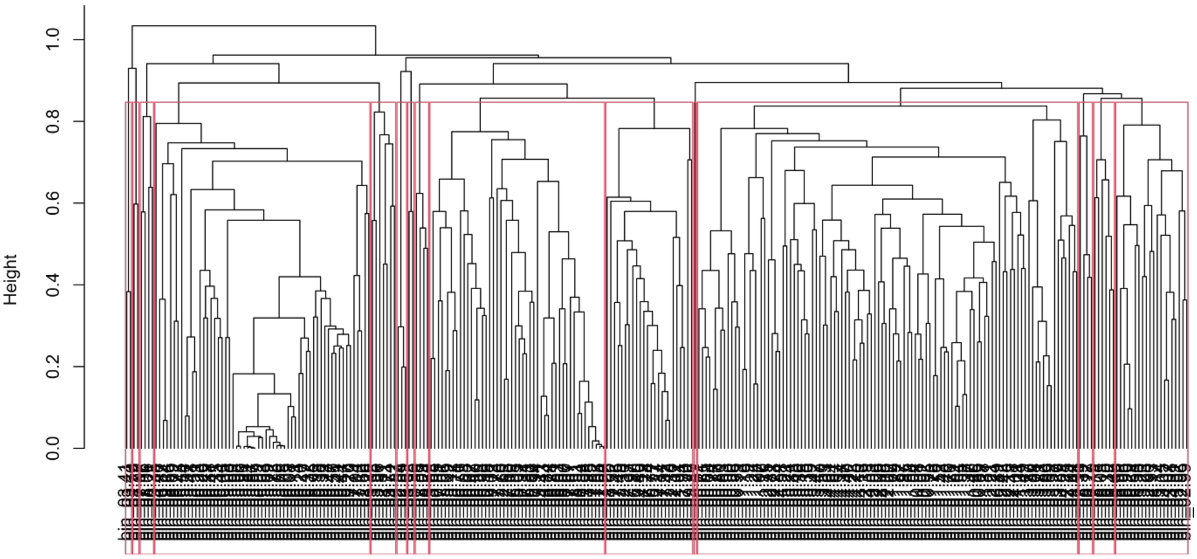

b

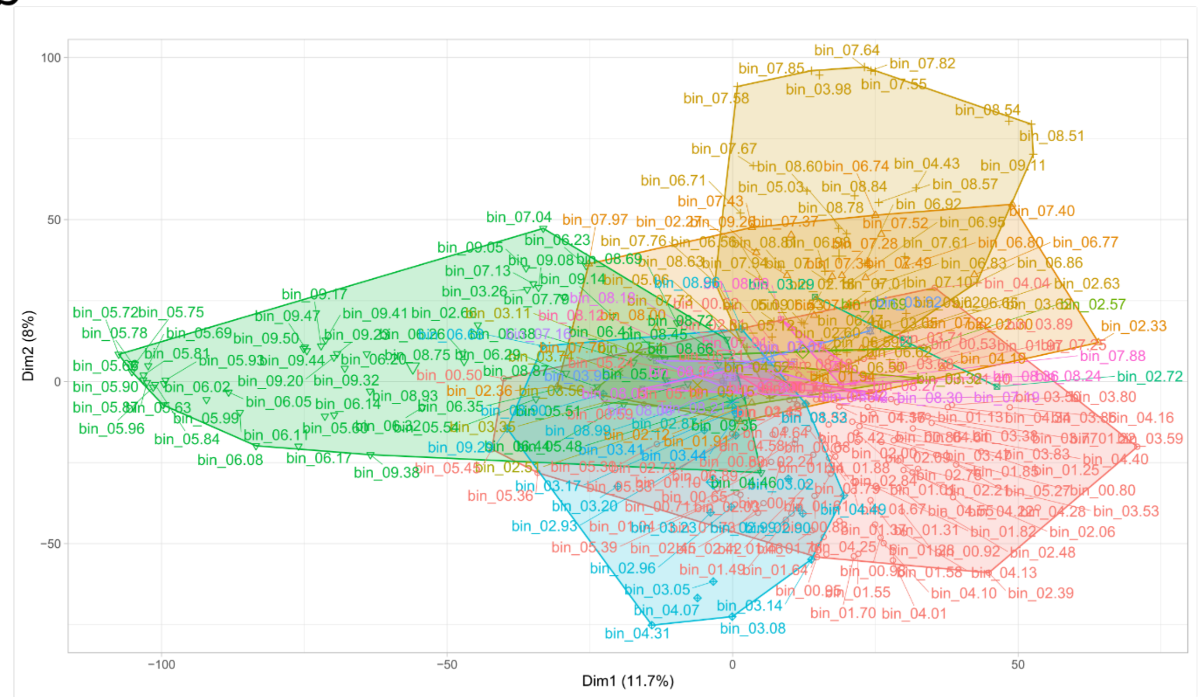

Figure S5. Clustering of urine bins. Panel a show a dendrogram generated from the hierarchical clustering analysis performed on urine bin values. The red rectangles represent the groups formed when the dendrogram tree is cut at a height of h=0.85. Those clusters are represented in panel b, which is a scores plot from a PCA of variables.

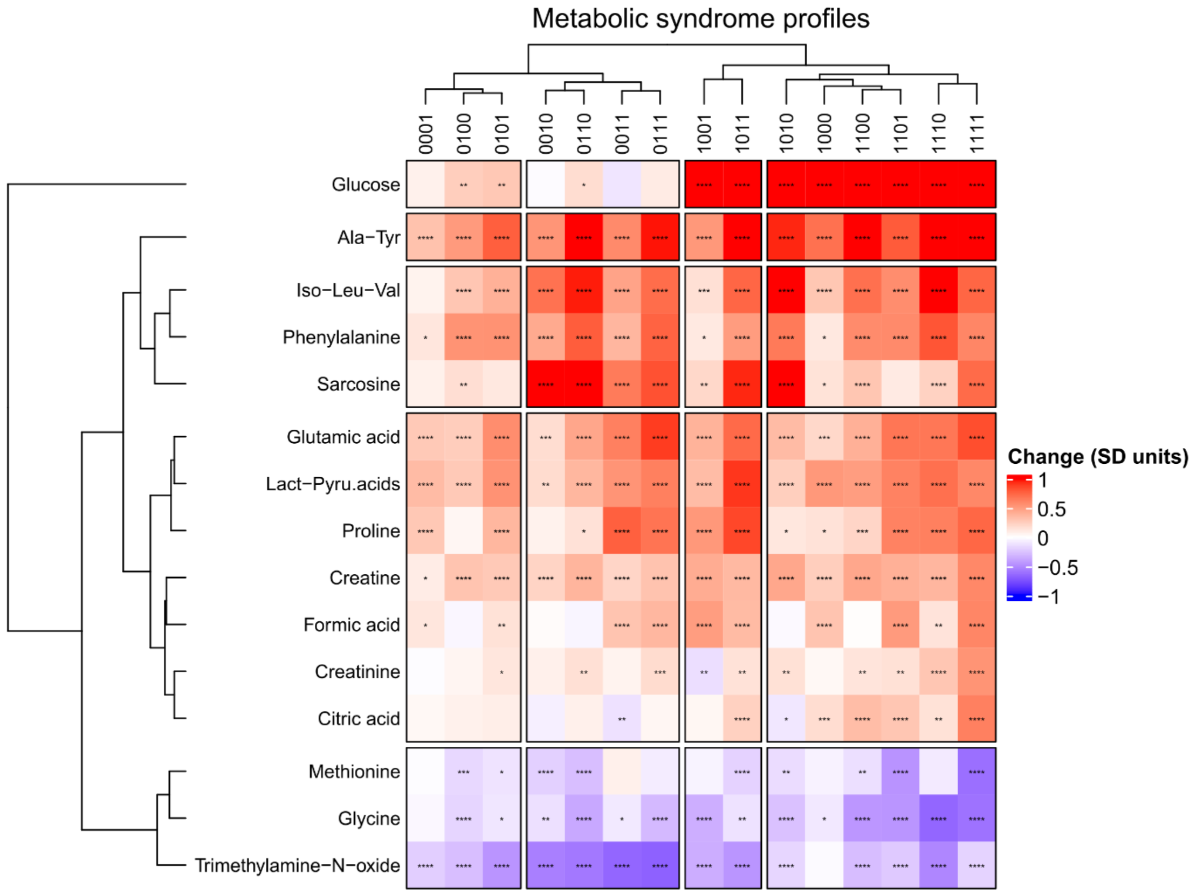

Figure S6. Heatmap representing the univariate analysis conducted for each metabolic syndrome profile compared to the asymptomatic profile for the metabo_serum dataset. The colors indicate the direction of change observed in the profile relative to the asymptomatic profile: red for positive and blue for negative. The intensity of the color refers to the amount of change in standard deviation units. If the change is statistically significant, it is indicated by asterisk symbols (*: adjusted p-value less than 0.05; **: p-value < 0.01; ***: p-value < 0.001; ****: p-value < 0.0001). Both the profiles and variables are clustered in a dendrogram obtained through hierarchical clustering.

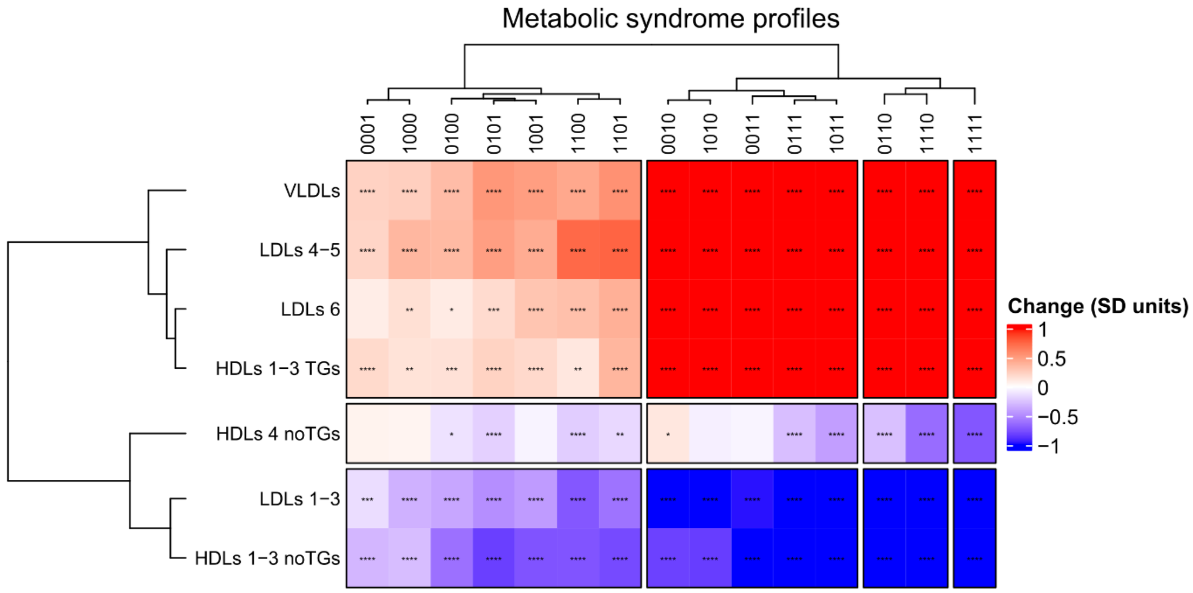

Figure S7. Heatmap representing the univariate analysis conducted for each metabolic syndrome profile compared to the asymptomatic profile for the lipo_serum dataset. The colors indicate the direction of change observed in the profile relative to the asymptomatic profile: red for positive and blue for negative. The intensity of the color refers to the amount of change in standard deviation units. If the change is statistically significant, it is indicated by asterisk symbols (*: adjusted p-value less than 0.05; **: p-value < 0.01; ***: p-value < 0.001; ****: p-value < 0.0001). Both the profiles and variables are clustered in a dendrogram obtained through hierarchical clustering.

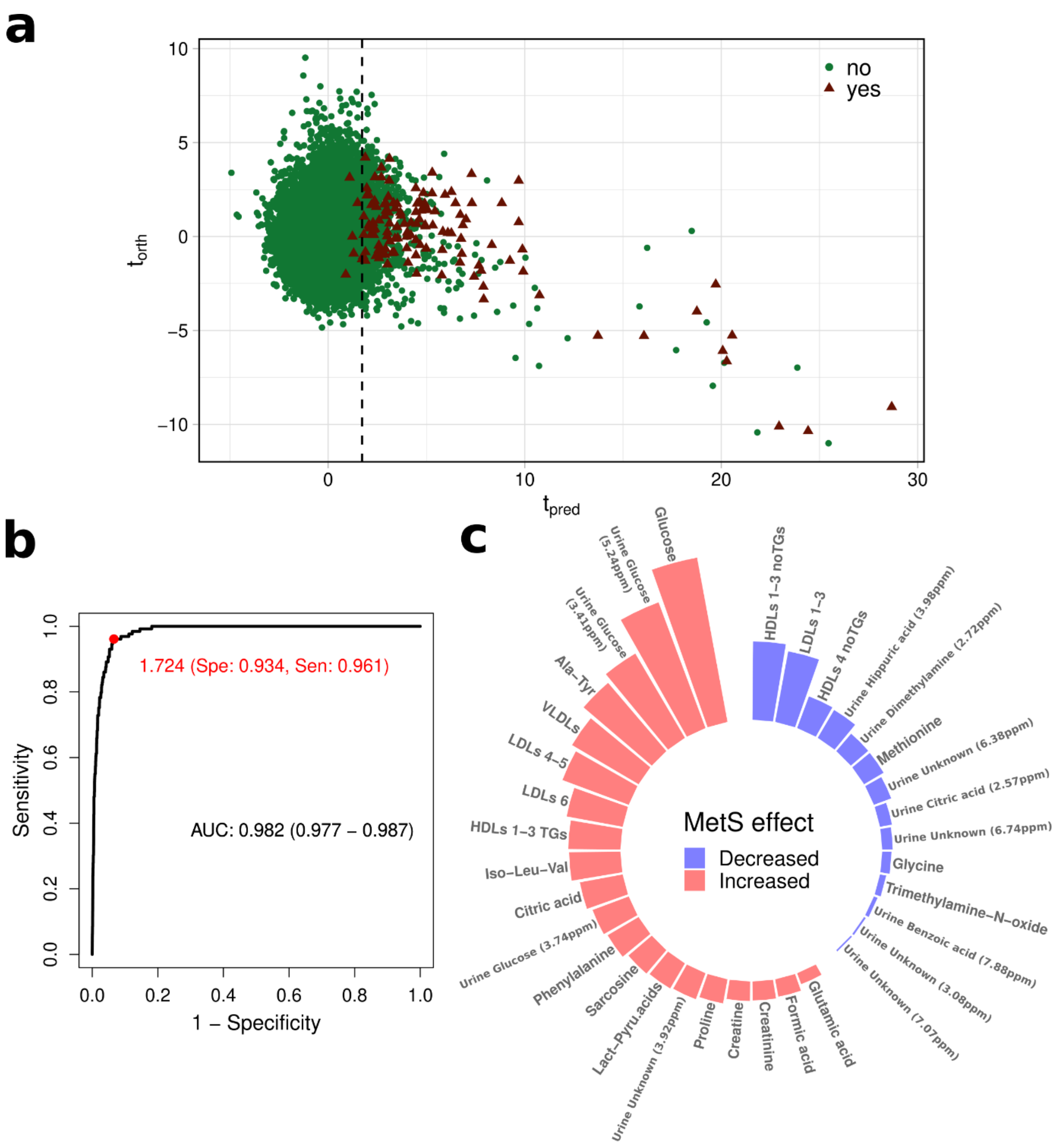

Figure S8. Metabolic syndrome model for the combined_serum/urine dataset based on O-PLS-DA combining urine and serum metabolomic data. a) scores plot with the predictive component on the X axis and the orthogonal component on the Y axis. The green dots represent individuals who do not have metabolic syndrome according to their metadata and WHO criteria, while the red triangles represent those who are classified as having metabolic syndrome. b) ROC curve showing the area under the curve for the final model along with its 95% confidence interval. It also indicates the sensitivity and specificity for the selected cutoff based on the Youden index. The dashed horizontal line shows the threshold selected using the Youden index from the ROC curve. c) Cartoon showing the most influential variables in the model; the size of the bar indicates their relative influence, while the color indicates whether they are elevated or not in metabolic syndrome.

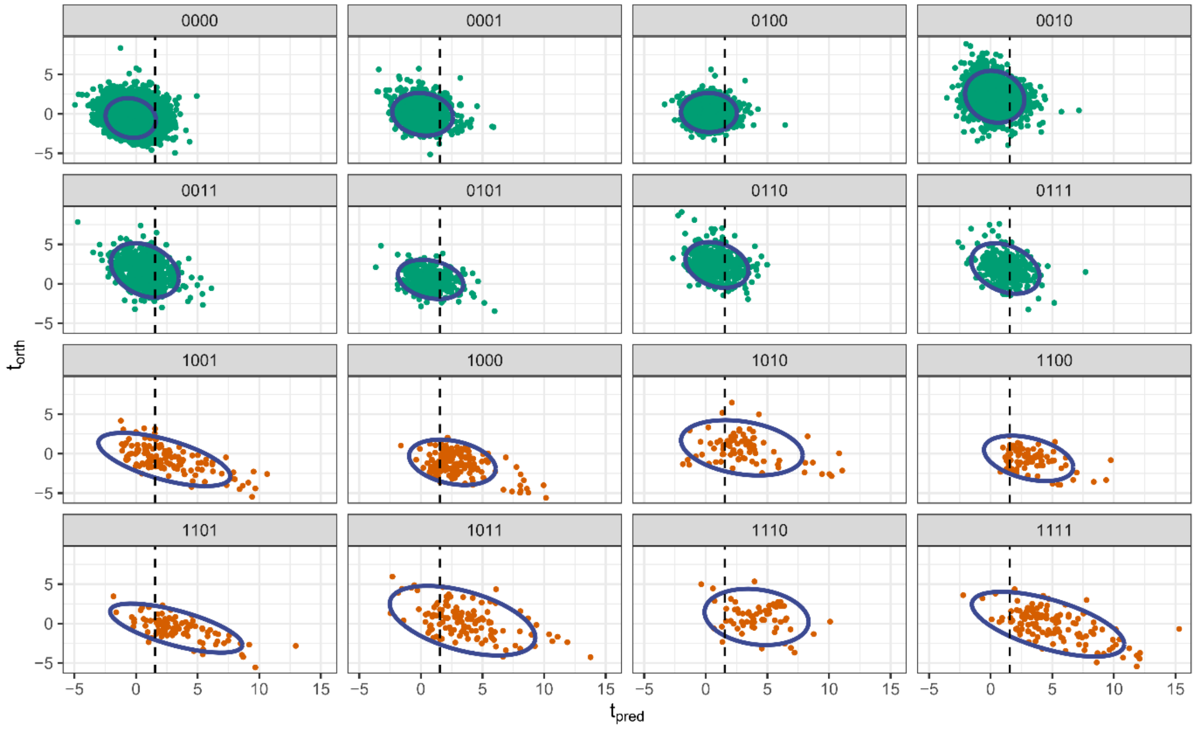

Figure S9. Projection of individuals from each metabolic syndrome profile onto the scores plot of the final O-PLS-DA metabo/lipo_serum model. The ellipse indicates the region where 95% of individuals are located. The profiles are ordered according to their average predictive component. Profiles with more than 50% of their individuals to the right of the vertical threshold are colored orange.

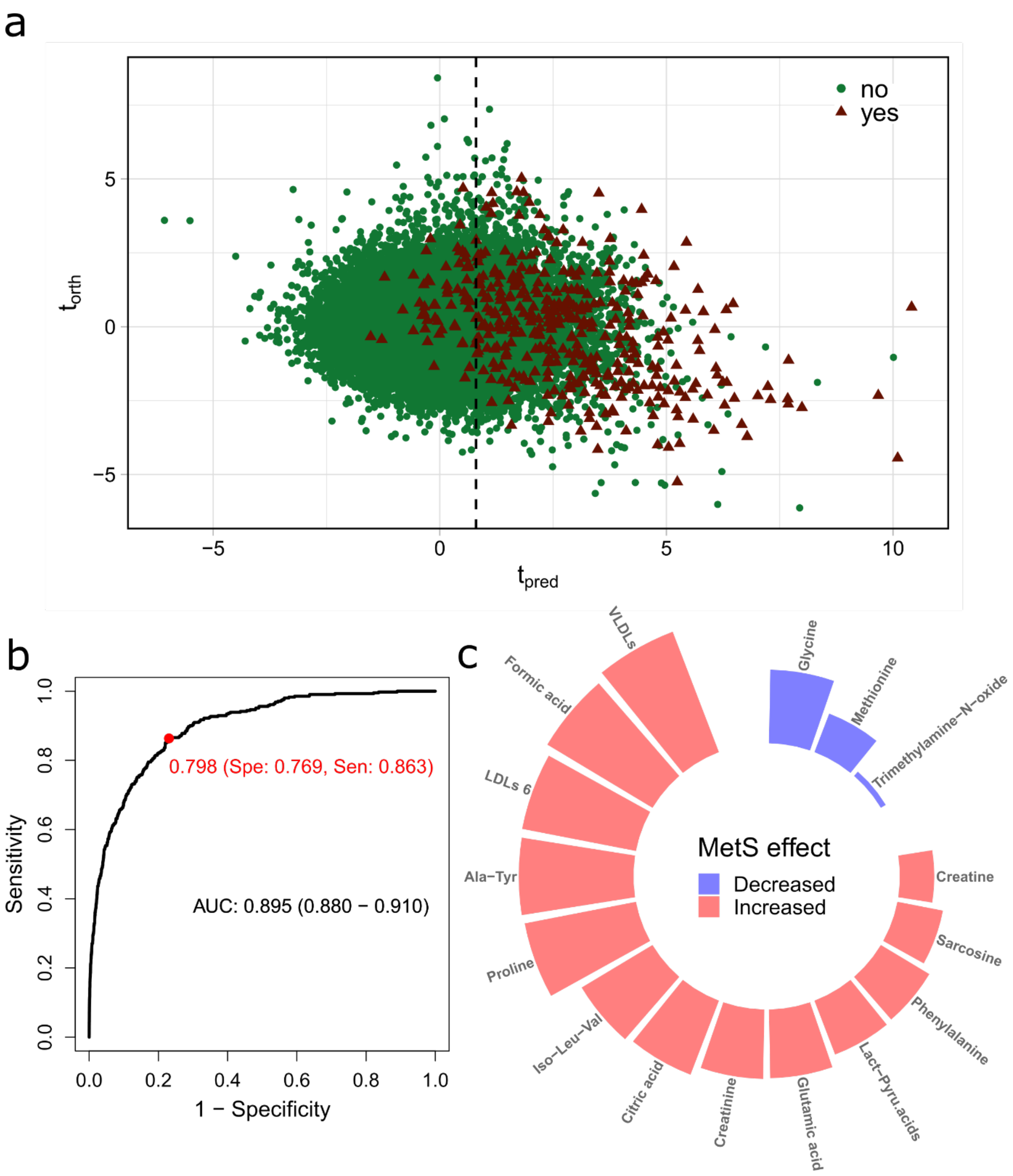

Figure S10. Metabolic syndrome model using the limited serum dataset: excluding Glucose, HDLs and LDLs. a) scores plot with the predictive component on the X axis and the orthogonal component on the Y axis. The green dots represent individuals who do not have metabolic syndrome according to their metadata and WHO criteria, while the red triangles represent those who are classified as having metabolic syndrome. b) ROC curve showing the area under the curve for the final model along with its 95% confidence interval. It also indicates the sensitivity and specificity for the selected cutoff based on the Youden index. The dashed horizontal line shows the threshold selected using the Youden index from the ROC curve. c) Cartoon showing the most influential variables in the model; the size of the bar indicates their relative influence, while the color indicates whether they are elevated or not in metabolic syndrome.

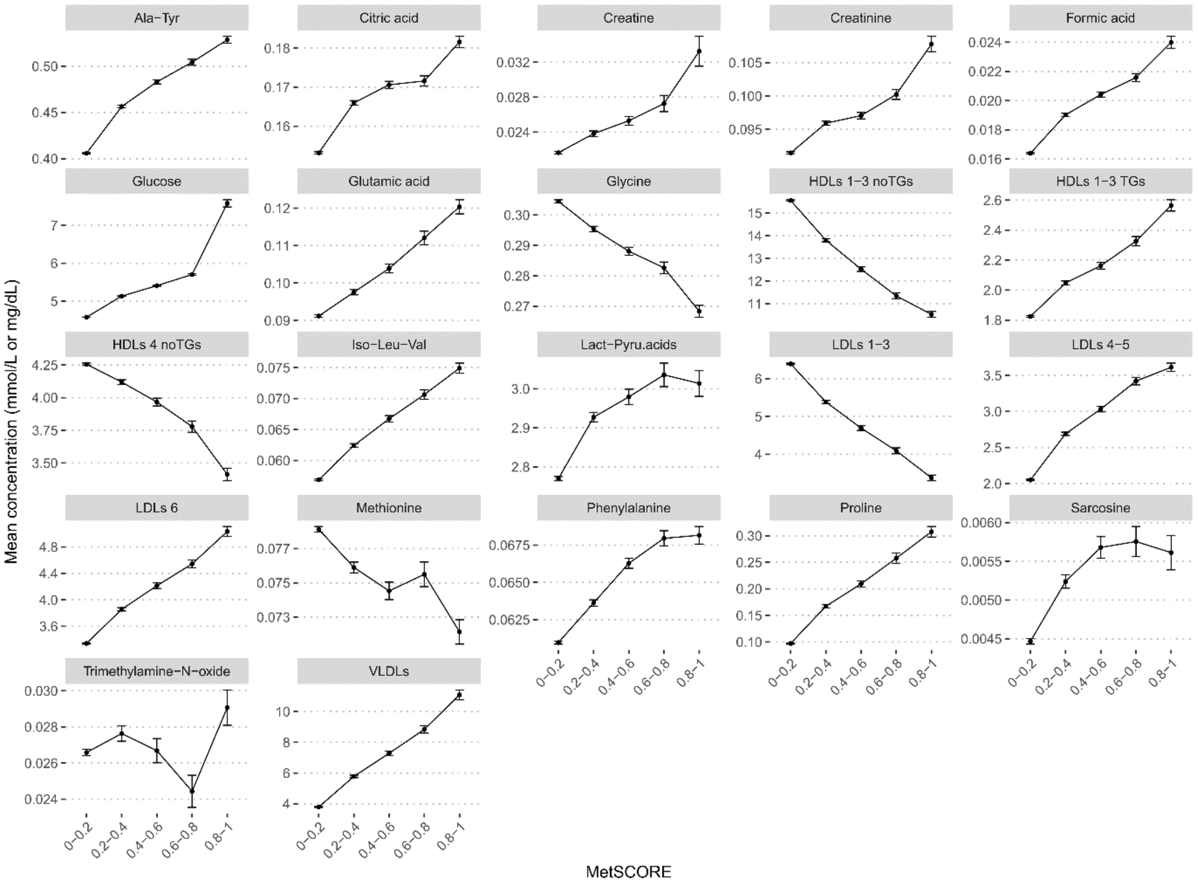

Figure S11. A plot with the evolution of the mean quantification values from the significant parameters of the MetSCORE. Evolution goes from low MetSCORE level (0-0.2) to high level (0.8-1). Each point represents the mean value, while standard errors are included as vertical lines. Quantification units are mmol/L for metabolites and mg/dL for lipoproteins.

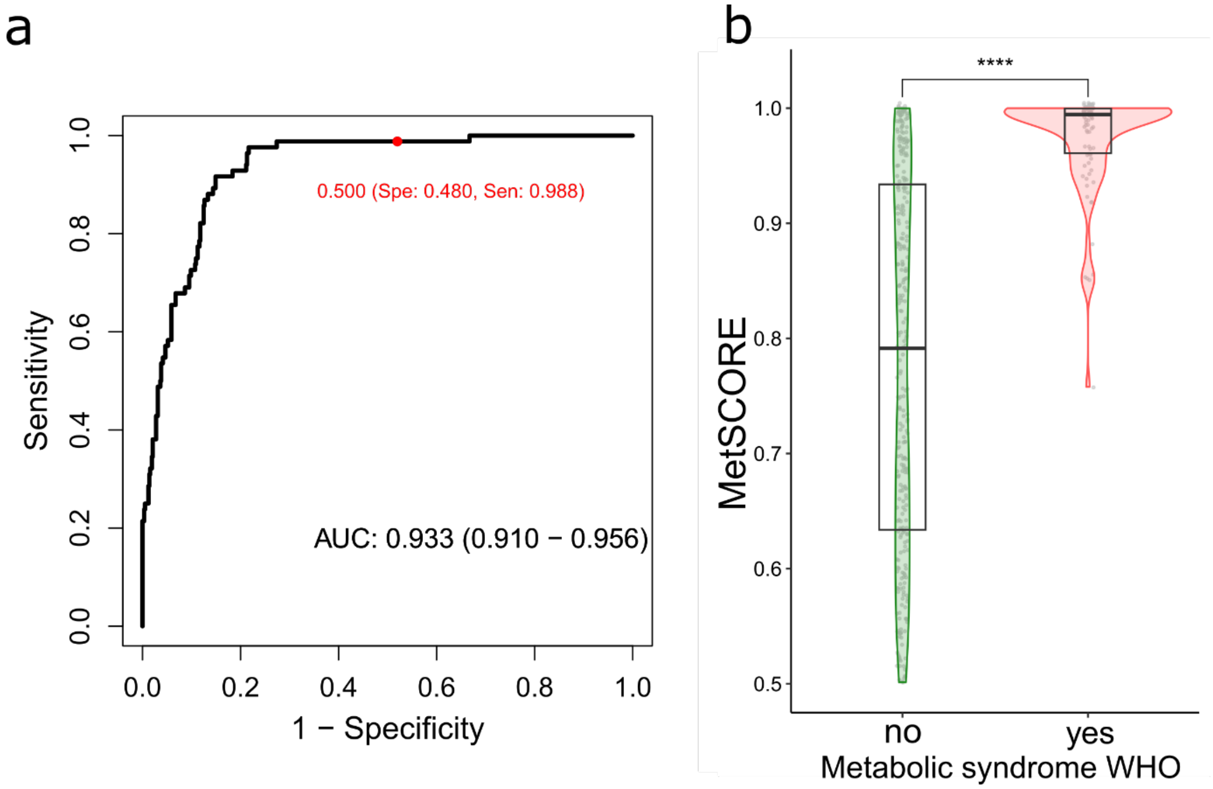

Figure S12. Performance of MetSCORE on the validation cohort. ROC curve in panel a show the area under the curve for the validation cohort along with its 95% confidence interval. It also indicates the sensitivity and specificity for the midpoint of the score. Panel b shows the distribution of those individuals with MetSCORE above 0.5; it is clearly shown that, despite the reduced specificity (at the expense of increasing sensitivity to nearly 100%), a significant difference is maintained between the values obtained by individuals classified as having metabolic syndrome by the WHO.

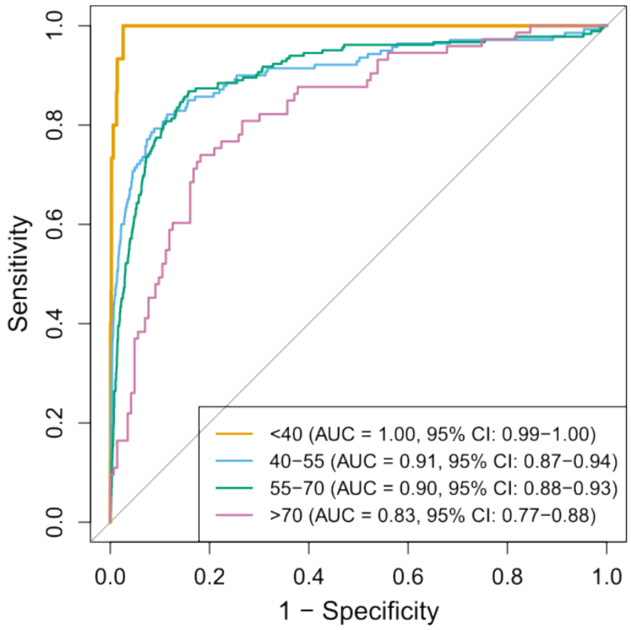

Figure S13. ROC curves of MetSCORE performance across different age groups. The figure shows the Receiver Operating Characteristic (ROC) curves for MetSCORE in four age groups: <40 years (orange), 40-55 years (blue), 55-70 years (green), and >70 years (pink). The AUROC values and 95% confidence intervals are indicated in the legend.

Supplementary references

1 Bizkarguenaga M, Gil-Redondo R, Bruzzone C, *et al.* Prospective Metabolomic Studies in Precision Medicine: The AKRIBEA Project. In: Handbook of Experimental Pharmacology. 2022: 275–97.

2 Bruzzone C, Bizkarguenaga M, Gil-Redondo R, *et al.* SARS-CoV-2 Infection Dysregulates the Metabolomic and Lipidomic Profiles of Serum. *iScience* 2020; **23**.

3 Cherubini A, Ostadreza M, Jamialahmadi O, *et al.* Interaction between estrogen receptor-α and PNPLA3 p.I148M variant drives fatty liver disease susceptibility in women. *Nature Medicine 2023 29:10* 2023; **29**: 2643–55.

4 Conde R, Oliveira N, Morais E, *et al.* NMR analysis seeking for cognitive decline and dementia metabolic markers in plasma from aged individuals. *J Pharm Biomed Anal* 2024; **238**: 115815.

5 Bruzzone C, Loizaga-Iriarte A, Sánchez-Mosquera P, *et al.* ^{1} H NMR-Based Urine Metabolomics Reveals Signs of Enhanced Carbon and Nitrogen Recycling in Prostate Cancer. *J Proteome Res* 2020; **19**: 2419–28.

6 Haudum CW, Kolesnik E, Colantonio C, *et al.* Cohort profile: 'Biomarkers of Personalised Medicine' (BioPersMed): a single-centre prospective observational cohort study in Graz/Austria to evaluate novel biomarkers in cardiovascular and metabolic diseases. *BMJ Open* 2022; **12**: e058890.

7 Kazenwadel J, Berezhnoy G, Cannet C, *et al.* Stratification of hypertension and SARS-CoV-2 infection by quantitative NMR spectroscopy of human blood serum. *Communications Medicine 2023 3:1* 2023; **3**: 1–15.

8 Kohonen T. Self-organized formation of topologically correct feature maps. *Biol Cybern* 1982; **43**: 59–69.

9 Kohonen T. Essentials of the self-organizing map. *Neural Networks* 2013; **37**: 52–65.

10 Beckonert O, Monnerjahn J, Bonk U, Leibfritz D. Visualizing metabolic changes in breast-cancer tissue using 1H-NMR spectroscopy and self-organizing maps. *NMR Biomed* 2003; **16**: 1–11.

11 Goodwin CR, Sherrod SD, Marasco CC, *et al.* Phenotypic mapping of metabolic profiles using self-organizing maps of high-dimensional mass spectrometry data. *Anal Chem* 2014; **86**: 6563–71.

12 Larmo PS, Kangas AJ, Soininen P, *et al.* Effects of sea buckthorn and bilberry on serum metabolites differ according to baseline metabolic profiles in overweight women: a randomized crossover trial. *Am J Clin Nutr* 2013; **98**: 941–51.

13 Weber A, Vivanco M d. M, Toca-Herrera JL. Application of self-organizing maps to AFM-based viscoelastic characterization of breast cancer cell mechanics. *Scientific Reports 2023 13:1* 2023; **13**: 1–10.

14 Nikolenko O, Labad F, Pujades E, *et al.* Combination of multivariate data analysis and mixing modelling to assess tracer potential of contaminants of emerging concern in aquifers. *Environmental Pollution* 2024; **341**: 123020.

15 Borkowska EM, Kruk A, Jedrzejczyk A, *et al.* Molecular subtyping of bladder cancer using Kohonen self-organizing maps. *Cancer Med* 2014; **3**. DOI:10.1002/cam4.217.

16 Trygg J, Wold S. Orthogonal projections to latent structures (O-PLS). *J Chemom* 2002; **16**: 119–28.

17 Bylesjö M, Rantalainen M, Cloarec O, Nicholson JK, Holmes E, Trygg J. OPLS discriminant analysis: combining the strengths of PLS-DA and SIMCA classification. *J Chemom* 2006; **20**: 341–51.

18 Kimhofer T, Lodge S, Whiley L, *et al.* Integrative Modeling of Quantitative Plasma Lipoprotein, Metabolic, and Amino Acid Data Reveals a Multiorgan Pathological Signature of SARS-CoV-2 Infection. *J Proteome Res* 2020; **19**: 4442–54.

19 Thévenot EA, Roux A, Xu Y, Ezan E, Junot C. Analysis of the Human Adult Urinary Metabolome Variations with Age, Body Mass Index, and Gender by Implementing a Comprehensive Workflow for Univariate and OPLS Statistical Analyses. *J Proteome Res* 2015; **14**: 3322–35.

20 Kang J, Guo X, Peng H, *et al.* Metabolic implications of amino acid metabolites in chronic kidney disease progression: a metabolomics analysis using OPLS-DA and MBRole2.0 database. *Int Urol Nephrol* 2023; : 1–12.

21 Holmes E, Loo RL, Stamler J, *et al.* Human metabolic phenotype diversity and its association with diet and blood pressure. *Nature 2008 453:7193* 2008; **453**: 396–400.

22 Guasch-Ferre M, Bhupathiraju SN, Hu FB. Use of Metabolomics in Improving Assessment of Dietary Intake. *Clin Chem* 2018; **64**: 82–98.
